# East-Asian *Helicobacter pylori* strains synthesize heptan-deficient lipopolysaccharide

**DOI:** 10.1371/journal.pgen.1008497

**Published:** 2019-11-20

**Authors:** Hong Li, Michael Marceau, Tiandi Yang, Tingting Liao, Xiaoqiong Tang, Renwei Hu, Yan Xie, Hong Tang, Alfred Tay, Ying Shi, Yalin Shen, Tiankuo Yang, Xuenan Pi, Binit Lamichhane, Yong Luo, Aleksandra W. Debowski, Hans-Olof Nilsson, Stuart M. Haslam, Barbara Mulloy, Anne Dell, Keith A. Stubbs, Barry J. Marshall, Mohammed Benghezal

**Affiliations:** 1 West China Marshall Research Center for Infectious Diseases, Center of Infectious Diseases, Division of Infectious Diseases, State Key Laboratory of Biotherapy, West China Hospital, Sichuan University, Chengdu, China; 2 Helicobacter pylori Research Laboratory, School of Biomedical Sciences, Marshall Centre for Infectious Disease Research and Training, University of Western Australia, Nedlands, Australia; 3 Univ. Lille, CNRS, Inserm, CHU Lille, Institut Pasteur de Lille, U1019—UMR 8204—CIIL—Center for Infection and Immunity of Lille, Lille, France; 4 Department of Life Sciences, Imperial College London, South Kensington Campus, London, United Kingdom; 5 Department of Gastroenterology, West China Hospital, Sichuan University, Chengdu, China; 6 Precision Medicine Key Laboratory of Sichuan Province & Precision Medicine Center, West China Hospital, Sichuan University, Chengdu, China; 7 Key Laboratory of Geoscience Spatial Information Technology, Ministry of Land and Resources of the P.R.China, Chengdu University of Technology; 8 School of Molecular Sciences, University of Western Australia, Crawley, Australia; 9 Ondek Pty Ltd, Rushcutters Bay, New South Wales, Australia; Università degli Studi di Milano, ITALY

## Abstract

The lipopolysaccharide O-antigen structure expressed by the European *Helicobacter pylori* model strain G27 encompasses a trisaccharide, an intervening glucan-heptan and distal Lewis antigens that promote immune escape. However, several gaps still remain in the corresponding biosynthetic pathway. Here, systematic mutagenesis of glycosyltransferase genes in G27 combined with lipopolysaccharide structural analysis, uncovered HP0102 as the trisaccharide fucosyltransferase, HP1283 as the heptan transferase, and HP1578 as the GlcNAc transferase that initiates the synthesis of Lewis antigens onto the heptan motif. Comparative genomic analysis of G27 lipopolysaccharide biosynthetic genes in strains of different ethnic origin revealed that East-Asian strains lack the *HP1283/HP1578* genes but contain an additional copy of *HP1105* and *JHP0562*. Further correlation of different lipopolysaccharide structures with corresponding gene contents led us to propose that the second copy of HP1105 and the JHP0562 may function as the GlcNAc and Gal transferase, respectively, to initiate synthesis of the Lewis antigen onto the Glc-Trio-Core in East-Asian strains lacking the *HP1283/HP1578* genes. In view of the high gastric cancer rate in East Asia, the absence of the *HP1283/HP1578* genes in East-Asian *H*. *pylori* strains warrants future studies addressing the role of the lipopolysaccharide heptan in pathogenesis.

## Author summary

The human gastric pathogen *Helicobacter pylori* is the most important aetiological factor for gastric cancer. *H*. *pylori* lipopolysaccharide, a major bacterial surface molecule, plays essential roles in host-pathogen interactions. Due to the scattered organisation of the lipopolysaccharide genes in its genome, several key enzymes involved in *H*. *pylori* lipopolysaccharide biosynthesis remain to be identified. Here, through systematic mutagenesis of glycosyltransferase genes in the model strain G27 combined with lipopolysaccharide structural analysis, we identified novel glycosyltransferases and established the first complete lipopolysaccharide biosynthetic pathway in G27. Furthermore, we analysed the conservation of the lipopolysaccharide genes across a large panel of *H*. *pylori* strains and demonstrated that many of the lipopolysaccharide genes are highly conserved, whereas the genes involved in lipopolysaccharide heptan incorporation are lacking in East-Asian strains. Finally, based on the correlation of lipopolysaccharide structure and gene contents in specific strains, we proposed a lipopolysaccharide biosynthetic model of how East-Asian strains, missing the heptan moiety, attach Lewis antigens onto the conserved Glc-Trio-Core. Future studies are needed to address whether the lack of heptan in lipopolysaccharide of East-Asian *H*. *pylori* strains is related to the high gastric cancer rate in East Asia, accounting for almost half of the worldwide gastric cancer cases.

## Introduction

*Helicobacter pylori* is a human gastric pathogen that infects more than half of the world’s population [1]. It causes active gastritis in all colonised subjects [2], and thus making it the most important aetiological factor for gastric cancer [2,3], the third leading cause of cancer related death worldwide [4]. Of note is that East Asia (China, Japan and Korea) alone accounts for more than half the worldwide gastric cancer cases [1,4], suggesting that the phylogeographic origin of *H*. *pylori* strains is implicated in gastric carcinogenesis [5].

**Fig 1:**
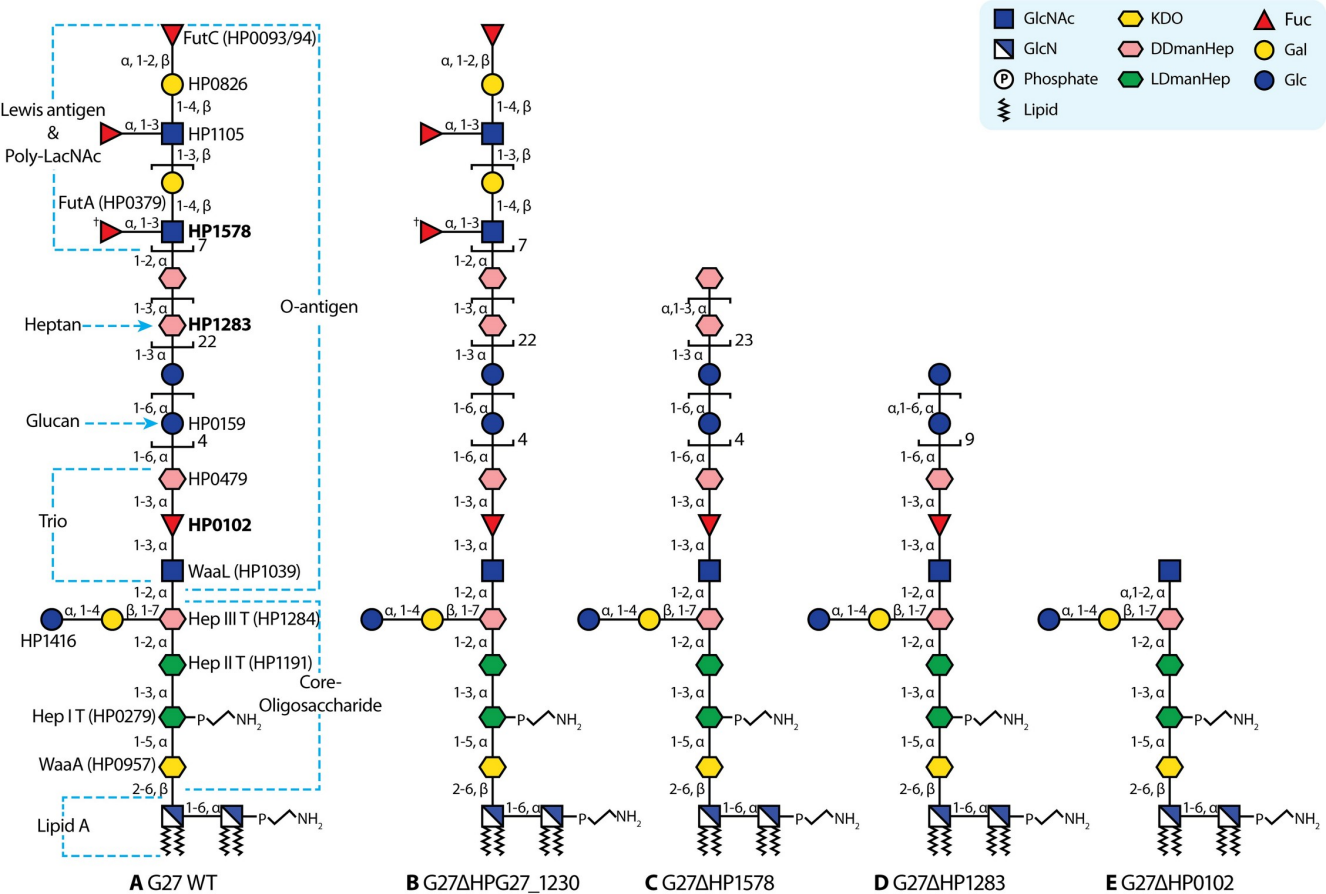
LPS structures in G27 wild-type and mutants. (**A**): Summary of glycosyltransferases underlying LPS biosynthesis in G27; (**B)**: G27Δ*HPG27_1230* LPS is similar to that of G27 wild-type; **(C)**: G27Δ*HP1578* LPS lacks the distal Lewis antigens but has a longer heptan (at least 24 repeating units); **(D)**: G27Δ*HP1283* LPS lacks the Lewis antigens and the heptan, but has a longer glucan (at least 9 repeating units); **(E)**: G27Δ*HP0102* LPS lacks the Lewis antigens, the heptan and the glucan. The O-antigen is not necessary composed of repeating poly Le^x^, it can also be poly-LacNAc with fewer Lewis epitopes. Glycosidic linkages are annotated from the non-reducing end.

The pathological outcomes of *H*. *pylori* chronic colonisation reflect the subtle host-pathogen interactions dictated by bacterial and host genetics and environmental factors. In this regard, *H*. *pylori* lipopolysaccharide (LPS), a major bacterial surface molecule, plays essential roles in host-pathogen interactions [6–9]. *H*. *pylori* LPS has three domains consisting of a hydrophobic lipid A domain embedded in the bacterial outer membrane (OM), a central core-oligosaccharide domain, and the outermost O-antigen [6,10]. Our group has recently elucidated the complete LPS structure in the *H*. *pylori* reference strain G27 and redefined the core-oligosaccharide domain as a hexasaccharide (Glc-Gal-DD-HepIII-LD-HepII-LD-HepI-KDO), which is decorated with a long O-antigen encompassing the trisaccharide (-DD-Hep-Fuc-GlcNAc-) termed as Trio, a glucan (homopolymer of Glc), a DD-heptan (homopolymer of Hep), and terminal Lewis antigens (**Fig 1A**) [6]. Compared to other Gram-negative bacteria, *H*. *pylori* constitutively synthesises an under-acylated and dephosphorylated lipid A, making it a poor ligand for Toll-like receptor 4, and conferring resistance to host cationic antimicrobial peptides [7]. As to the function of the O-antigen domain, its mimicry of host Lewis antigens leads to suppression of the proinflammatory response through O-antigen binding to the DC-SIGN receptor to regulate dendritic cell function [11]. The involvement of the LPS core-oligosaccharide domain in host-pathogen interactions comes from the very recent identification of ADP-DD/LD-Hep as a novel pathogen associated molecular pattern (PAMP) [12–16]. ADP-LD-Hep is the precursor of the LD-Hep units conservatively present in the LPS core-oligosaccharide of nearly all Gram-negative bacteria [17]. However, the incorporation of DD-Hep into bacterial LPS is rare. The receptor for ADP-DD/LD-Hep is the host ALPK1 (alpha-kinase1) that upon binding activates TIFA (TRAF-interacting protein with forkhead-associated domain)-dependent NF-κB-mediated inflammatory response in the host cytosol [12]. In *H*. *pylori*, the stimulation of the ALPK1-TIFA axis signalling pathway is dependent on the *cag* type 4 secretion system (CagT4SS) [13–15]. Intriguingly, one of the unique features of *H*. *pylori* LPS is the presence of both LD- and DD-Hep units in the core-oligosaccharide domain, and also a common occurrence of the intervening DD-heptan in Western *H*. *pylori* strains (26695 and G27 as examples) [10,18]. In contrast, only one study to date has analysed the LPS structures of East Asian strains, and none of the structures displayed the DD-heptan moiety, despite the presence of Lewis antigens [19].

In view of the essential roles played by *H*. *pylori* LPS in host-pathogen interactions, and the LPS structural variations observed between Western and East Asian *H*. *pylori* strains, we hypothesized distinct differences in LPS glycosyltransferase gene content among *H*. *pylori* strains of different phylogeographic origin, and their implications in host-pathogen interactions and carcinogenesis. Here, using a combined approach of genetics, bioinformatics, and structural analyses, we identified missing LPS glycosyltransferase genes in G27 and propose a *H*. *pylori* LPS biosynthetic model that accounts for the different LPS structures expressed by strains of different phylogeographic origin.

## Results

### Genome-wide identification of LPS glycosyltransferase genes in *H*. *pylori* strain G27

In order to analyse LPS gene content among *H*. *pylori* strains of different phylogeographic origin, a complete LPS gene set in a single strain was required as a reference. However, glycosyltransferases involved in the assembly of the core-oligosaccharide and O-antigen domains have not been fully identified, which is possibly due to the scattered organisation of LPS biosynthetic genes in the *H*. *pylori* genome. Thus, the first goal of this study was to identify the complete set of LPS glycosyltransferase genes in the *H*. *pylori* reference strain G27. This strain is fully sequenced and has been extensively used for *H*. *pylori* research [20], and its complete LPS structure has been recently elucidated [6].

To identify the complete LPS glycosyltransferase gene set in G27, a genome-wide search of glycosyltransferase genes in this strain was conducted using the Carbohydrate-Active Enzymes (CAZy) database [21], which enabled the identification of 24 glycosyltransferase genes (**Table 1**). For nomenclature reasons, gene names of orthologs in the reference strain 26695 were used throughout this study unless the genes were absent in the 26695 genome, in this case gene names of strain G27 were used.

**Table 1:** CAZy family assignment of *H*. *pylori* glycosyltransferase genes.

NO.	GT family	26695 ORF	G27 ORF	Activity	Function	Mutatagenesis in this study	Reference(s)
1.	GT-2	HP0102	HPG27_94	Putative glycosyltransferase	Unknown	√	this study
2.	GT-8	HP1578	HPG27_1515	Putative glycosyltransferase	Unknown	√	this study
3.	GT-9	※	HPG27_1229	Putative glycosyltransferase	Unknown		this study
4.	GT-9	※	HPG27_1230	Putative glycosyltransferase	Unknown	√	this study
5.	GT-25	HP0805	HPG27_761	Putative glycosyltransferase	Unknown	√	this study
6.	GT-9	HP1283	HPG27_1235	Putative heptosyltransferase^**#**^	Heptan^#^	√	this study and [22]
7.	GT-19	HP0867	HPG27_821	LpxB	Lipid A		[10]
8.	GT-30	HP0957	HPG27_905	WaaA			[10]
9.	GT-9	HP0279	HPG27_258	Hep I transferase	Core-oligosaccharide	√	[6]
10.	GT-9	HP1191	HPG27_1136	Hep II transferase		√	[6]
11.	GT-9	HP1284	HPG27_1236	Hep III transferase		√	[6]
12.	GT-8	HP1416	HPG27_ 1339	α-1,2/4 Glc transferase		√	[23]
13.	GT-9	HP0479	HPG27_437	α-1,2-DD-Hep transferase	Trio	√	[24,25]
14.	GT-8	HP0159	HPG27_146	α-1,6 Glc transferase	Glucan	√	[23]
15.	GT-8	HP1105	HPG27_1046	β-1,3-GlcNAc transferase	Lewis antigen	√	[26]
16.	GT-25	HP0619 (JHP0562/3)	HPG27_579/580	β-1,3-Gal transferase		√	[27–29]
17.	GT-25	HP0826	HPG27_785	β-1,4-Gal transferase		√	[30]
18.	GT-10	HP0379	HPG27_613	FutA		√	[31]
19.	GT-10	HP0651	HPG27_1018	FutB		√	[31]
20.	GT-11	HP0093/0094	HPG27_86	FutC		√	[32]
21.	GT-8	HP0208	HPG27_190	Putative glycosyltransferase	LPS biosynthesis ?	√	[26,33]
22.	GT-4	HP0421	HPG27_952	Cholesterol α-glucosyltransferase	Not associated with LPS biosynthesis		[34]
23.	GT-28	HP1155	HPG27_1099	MurG			[10]
24.	GT-51	HP0597	HPG27_557	PBP-1A			[10]

※ partial fragment or homologue not found in this strain

# *HP1283* function was unknown at the time of this study but has recently been annotated [22]

Of the 24 CAZy-annotated glycosyltransferase genes, more than half of them were previously known to be involved in *H*. *pylori* LPS biosynthesis and were mapped onto the complete G27 LPS structure (**Fig 1A** and **Table 1**). Of note, although not being mapped onto G27 LPS structure, *HPG27_579* and *HPG27_580* were found to be split genes of *HP0619*, a pseudogene in 26695. *HPG27_579* and *HPG27_580* are homologous to *JHP0562* and *JHP0563 i*n strain J99. *JHP0563* encodes a β-1,3-Gal transferase, which was reported to be essential for the production of type 1 Lewis antigens (Le^a^ and Le^b^) [27,28]. Interestingly, the mutagenesis of *JHP0562*, present in many but not all *H*. *pylori* strains, results in the loss of both type 1 and type 2 Lewis antigen expression [27–29]. HP0208 was not mapped onto the G27 LPS structure either, but it has also been suggested to play a role in LPS biosynthesis [33].

Of the 24 CAZy-annotated glycosyltransferase genes, six had not been previously studied and were likely to be the missing LPS glycosyltransferase genes in G27: *HP0102* (*HPG27_94*), *HP1578* (*HPG27_1515*), *HPG27_1229*, *HPG27_1230*, *HP0805* (*HPG27_761*) and *HP1283* (*HPG27_1235*) (**Fig 1A** and **Table 1**). Of note, the identity of *HP1283* was unknown at the time of this study but has recently been reported to encode the heptan transferase [22]. *HPG27_1229* was found to be a partial *HP1284*, and therefore was not considered to be a functional glycosyltransferase gene.

### Systematic mutational analysis of LPS Genes in *H*. *pylori* strain G27

To obtain a complete set of LPS gene mutants in G27, we conducted a systematic mutagenesis of all the known and putative LPS glycosyltransferase genes in G27 with the exclusion of five glycosyltransferase genes: *HP0421*, the cholesterol α-glucosyltransferase gene [34]; *HP1155* and *HP0597*, the glycosyltransferase genes involved in peptidoglycan biosynthesis [35]; *HP0867* and *HP0957*, the essential glycosyltransferase genes involved in KDO_2_-lipid A biosynthesis [10] (**Table 1)**. Additionally, the other three enzymes WecA (HP1581), Wzk (HP1206) and WaaL (HP1039) involved in the O-antigen initiation, translocation and ligation, respectively, were also included for mutagenesis to allow for better comparison of LPS phenotypes [36].

**Fig 2:**
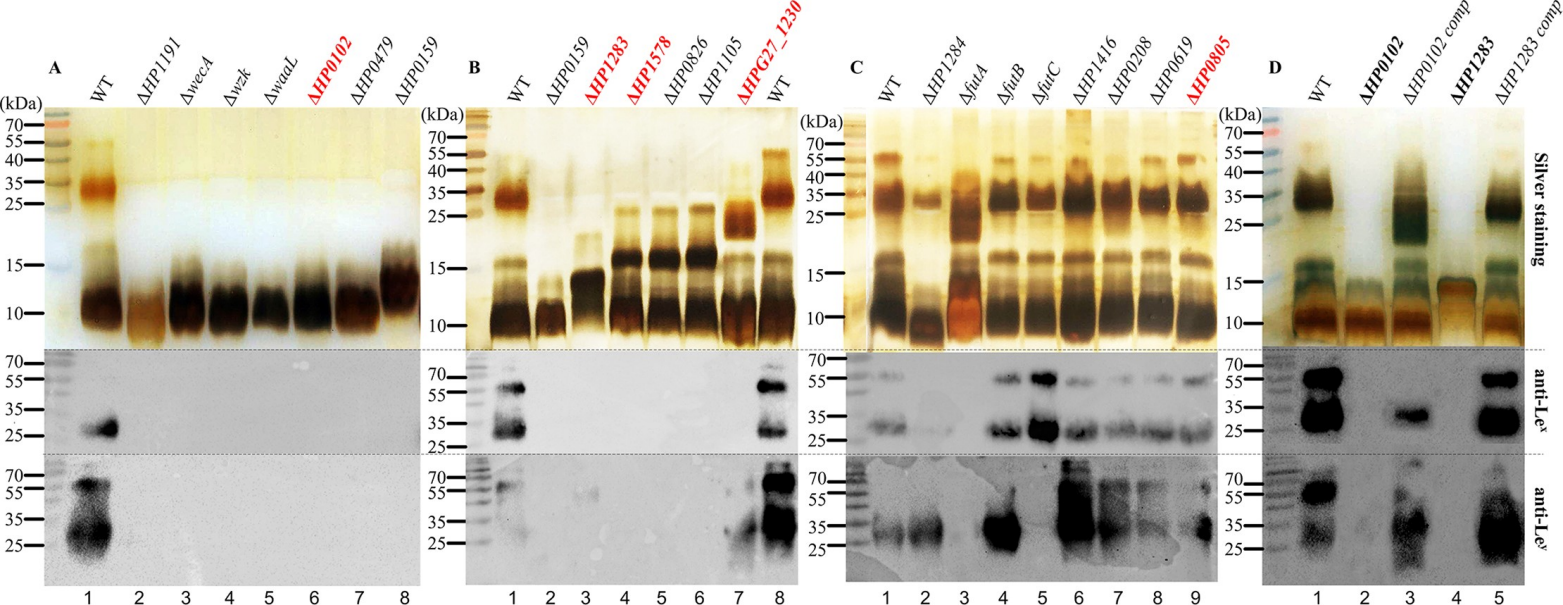
Systematic mutational analysis of LPS genes in G27. LPS samples from G27 wild-type and isogenic LPS mutants were analysed by silver staining (upper panel), and Western blot using anti-Le^x^ (middle panel) and anti-Le^y^ antibodies (lower panel). (**A**): Lane 1: G27 wild-type full-length LPS expressing both Le^x^ and Le^y^; Lane 2: the Hep II transferase mutant Δ*HP1191*; Lane 3–5: the O-antigen initiating enzyme (WecA), flippase (Wzk), and ligase (WaaL) mutants; Lane 6: the new glycosytransferase gene mutant Δ*HP0102*; Lane 7: the Trio Hep transferase mutant Δ*HP0479*; Lane 8: the glucan transferase mutant Δ*HP0159*; (**B**): Lane 1–2: G27 wild-type and Δ*HP0159*; Lane 3–4: the new glycosytransferase gene mutants Δ*HP1283* and Δ*HP1578*; Lane 5–6: the poly-LacNAc Gal and GlcNAc transferase mutants Δ*HP0826* and Δ*HP1105*; Lane 7: the new glycosytransferase gene mutant Δ*HPG27_1230*; Lane 8: G27 wild-type; (**C**): Lane 1–9: G27 wild-type, the Hep III transferase mutant Δ*HP1284*, Δ*futA*, Δ*futB*, Δ*futC*, Δ*HP1416*, Δ*HP0208*, Δ*HP0619*, and the new glycosyltransferase mutant Δ*HP0805*; (**D**): Lane 1, G27 wild-type; Lane 2–3, Δ*HP0102* and Δ*HP0102* complementation; Lane 4–5, Δ*HP1283* and Δ*HP1283* complementation.

Using the Xer-cise gene deletion technique developed by our group [37], all the selected LPS genes except the essential Hep I transferase gene *HP0279* [38], were successfully deleted in the single genetic background G27. Subsequently, LPS samples isolated from G27 wild-type and the isogenic mutants were resolved on SDS-PAGE for comparison of LPS length by silver staining and of Lewis antigen expression by Western blot. Apparent LPS truncation was observed in 11 mutants with LPS length increasing in the following order G27Δ*HP1191* < G27Δ*wecA* ≈ G27Δ*wzk* ≈ G27Δ*waaL* ≈ G27Δ*HP0102* ≈ G27Δ*HP0479* < G27Δ*HP0159* < G27Δ*HP1283* < G27Δ*HP1578* ≈ G27Δ*HP0826* ≈ G27Δ*HP1105* < G27 wild-type (**Fig 2A and 2B**). G27 wild-type expressed Le^x^ and Le^y^, whereas the 11 mutants were negative for both Le^x^ and Le^y^ (**Fig 2A and 2B**). The observed LPS truncation and loss of Lewis antigen expression confirmed the involvement of *HP1191*, *wecA*, *wzk*, *waaL*, *HP0479*, *HP0159*, *HP0826* and *HP1105* in G27 LPS biosynthesis (**Fig 2A and 2B** and **Fig 1A**).

The deletion of *HPG27_1230* resulted in a slight change to the LPS profile and the loss of Le^x^ (**Fig 2B**). As expected, G27Δ*HP1284* LPS displayed a loss of bands sized around 15–20 kDa LPS (**Fig 2C**), which is due to the lack of Hep III and the attached disaccharide [6]. The deletion of *futA* (*HP0379*), *futB* (*HP0651*) and *futC* (*HP0093/94)* in G27 had different effects on Le^x/y^ expression (**Fig 2C**). G27Δ*futA* was negative for both Le^x^ and Le^y^, whereas G27Δ*futB* expressed both Le^x^ and Le^y^ (**Fig 2C**), suggesting that G27 FutA has a α-1,3 FucT activity required for the generation of both epitopes, whereas FutB in G27 is not required for Le^x/y^ generation. FutC is a α-1,2 FucT which adds a second Fuc residue to Le^x^ to generate Le^y^ [32], and as expected G27Δ*futC* was positive for Le^x^ expression only (**Fig 2C**). G27Δ*HP1416*, G27Δ*HP0208*, G27Δ*HP0619* and G27Δ*HP0805* displayed LPS length like wild-type G27, and all expressed both Le^x^ and Le^y^ (**Fig 2C**).

Genetic complementation of G27Δ*HP0102* and G27Δ*HP1283* restored the full-length LPS (**Fig 2D**). Complementation of G27Δ*HP1283* restored the expression of both Le^x^ and Le^y^, whereas complementation of G27Δ*HP0102* restored the expression of Le^y^ only (**Fig 2D**). Genetic complementation of G27Δ*HP1578* was unsuccessful as no clone could be recovered after multiple conjugation attempts, which may be due to the low efficiency of the conjugation method, or due to a second-site mutation.

Collectively, the change of LPS profiles observed in G27Δ*HP0102*, G27Δ*HP1283*, G27Δ*HP1578* and G27Δ*HPG27_1230* provides evidence that the HP0102, HP1283, HPG27_1230 and HP1578 are likely to be novel glycosyltransferases involved in G27 LPS biosynthesis.

### LPS structural characterisation enabled the identification of the missing LPS glycosyltransferase genes in G27

**Fig 3:**
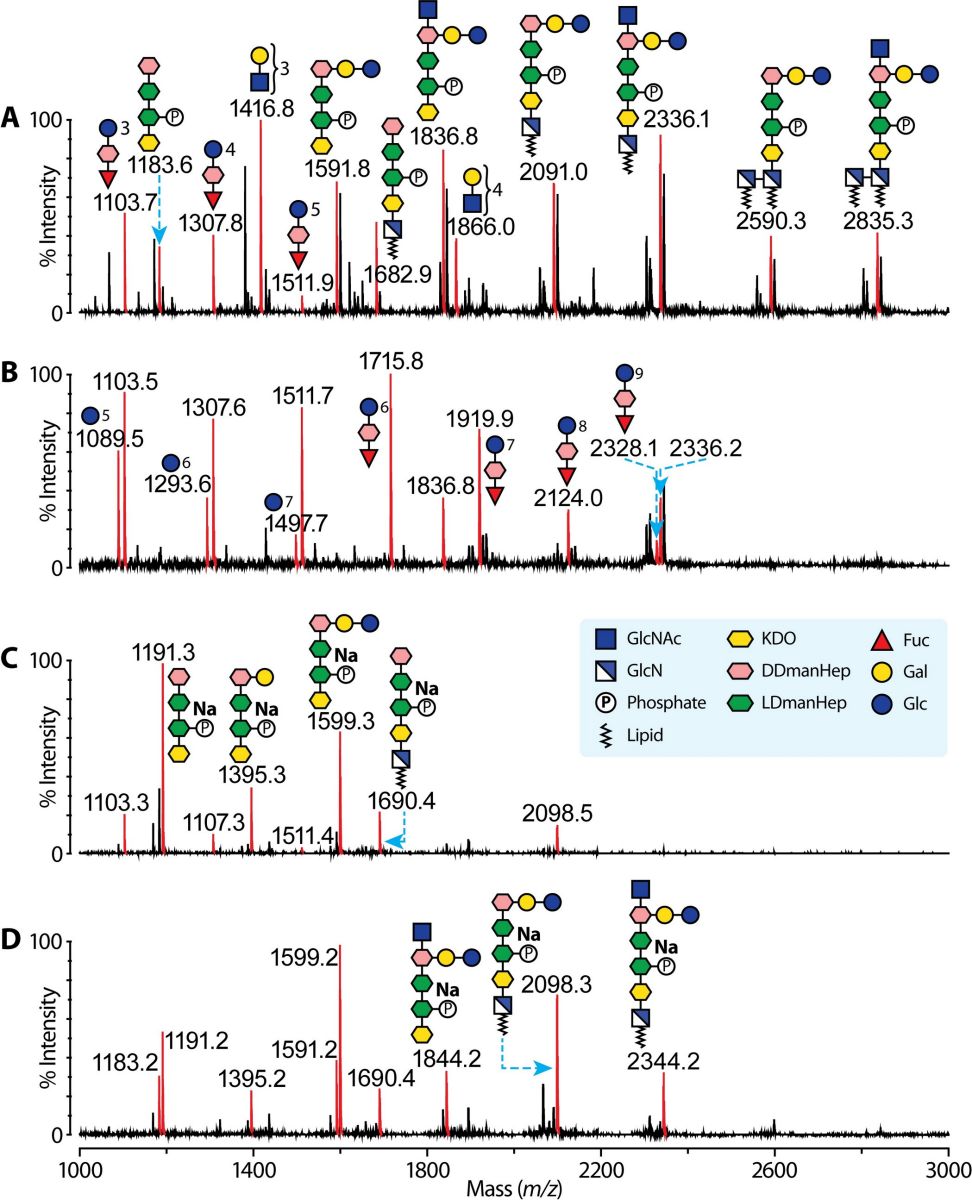
MS analysis of G27 mutant LPS. MALDI-TOF spectra of (**A**): Δ*HPG27_1230*; (**B**): Δ*HP1283*; (**C**): Δ*HP1578* and (**D**): Δ*HP0102* LPS after methanolysis and permethylation. Red peaks corresponding to sodiated permethylated glycans are annotated with mass-to-charge ratio and glycan structures. MS signals are only annotated once, *i*.*e*., signals observed in spectrum (**A**) are not annotated again in spectrum (**B**), (**C**) and (**D**). Note that some peaks in the spectrum corresponding to the core region exhibit 8 Da satellite peaks when compared with Fig 3A, due to under-permethylation. Some of these peaks are not annotated in the figure. The observed MS signals are strongly indicative of several types of glycan structures, including poly-LacNAc (*m/z* 1416.8 and 1866.0), a glucan (*m/z* 1103.7, 1307.8 and 1511.9), core-oligosaccharide (*m/z* 1183.6, 1591.8, 1836.8) and core-lipid A (*m/z* 2091.0. 2336.1, 2590.3 and 2835.3). The poly-LacNAc and glucan are methanolysed from the LPS O-antigen and the remaining signals are from core-oligosaccharide and Lipid A. It is evident from the data that LPS from all four mutants share the same core-lipid A structure. Only the Δ*HPG27_1230* LPS possesses the poly-LacNAc, and its structure is very similar to previously characterised G27 wild-type LPS structure. The Δ*HP1283* LPS has an elongated glucan. Δ*HP1578* LPS has a glucan of five repeating units, while the Δ*HP0102* LPS has no glucan. The Δ*HP1578* LPS was further characterised by mild HF hydrolysis in S1 Fig.

To assign each of these above newly discovered glycosyltransferase genes onto G27 LPS biosynthesis, the LPS structures from corresponding mutants were elucidated. LPS isolated from G27Δ*HPG27_1230*, G27Δ*HP1283*, G27Δ*HP1578* and G27Δ*HP0102* was analysed using previously published methanolysis and MS methods [6]. Matrix-assisted laser desorption/ionization time of flight (MALDI-TOF) mass fingerprints of the methanolysed LPS glycans after permethylation are shown in **Fig 3**. The annotation of MS peaks was based on the previously characterised LPS from strains 26695 [25] and G27 [6]. Most un-annotated peaks are due to incomplete permethylation of phosphorylated glycans.

The MS data indicate G27Δ*HPG27_1230* LPS (**Fig 3A**) is similar to G27 wild-type LPS. Its O-antigen contains poly-LacNAc (*m/z* 1416.8 and 1866.0) and a glucan (*m/z* 1103.7, 1307.8 and 1511.9) that can be as long as five Glc units. MS peaks corresponding to core-oligosaccharide (*m/z* 1183.6, 1591.8, 1836.8) and core-lipid A (*m/z* 1682.9, 2091.0. 2336.1, 2590.3 and 2835.3) were observed, which strongly indicates G27Δ*HPG27_1230* LPS has the hexasaccharide core (Glc-Gal-tri-Hep-KDO) like G27 wild-type LPS (**Fig 1B**).

G27Δ*HP1283* LPS carries an elongated glucan (m/z 1089.5, 1103.5, 1293.6, 1307.6, 1497.7, 1511.7, 1715.8. 1919.9, 2124.0 and 2328.1) that contains at least 9 Glc units (**Fig 3B**). No poly-LacNAc was found in G27Δ*HP1283* LPS, whereas the core-lipid A region is conserved (*m/z* 1836.8 and 2336.2). The MS data indicate HP1283 is a heptosyltransferase that caps the glucan, and therefore its mutation leads to glucan elongation as reported in a previous study (**Fig 1D**) [22].

G27Δ*HP1578* LPS carries a normal glucan (*m/z* 1103.3, 1307.3 and 1511.4) and core-oligosaccharide (*m/z* 1191.3, 1395.3, 1599.3, 1690.4 and 2098.5) (**Fig 3C**). G27Δ*HP0102* LPS gives the simplest MS pattern (**Fig 3D**), in which most signals are derived from the core-lipid A region. The MS peaks at *m/z* 1844.2 and 2344.2 and the absence of the glucan peaks indicate the core-oligosaccharide is only capped with a single GlcNAc. This observation suggests that HP0102 is the fucosyltransferase involved in the biosynthesis of the Trio (Hep-Fuc-GlcNAc) that links core-oligosaccharide and the rest of the O-antigen (**Fig 1E**).

The LPS samples from G27Δ*HPG27_1230* and G27Δ*HP1578* were further subjected to Smith degradation and mild HF hydrolysis (**S1 Fig**). The MALDI-TOF spectrum of mildly oxidised G27Δ*HPG27_1230* LPS (**S1A Fig**) shows two clusters of MS peaks, *i*.*e*., MS peaks at *m/z* 534,3, 983.6 and 1423.8 corresponding to poly-LacNAc and MS peaks at *m/z* 779.5, 1228.7, 1677.9, 2127.1 2576.2, 3025.3 and 3474.3 corresponding to GlcNAc-poly-LacNAc. The longest observed poly-LacNAc has 8 repeating units. Overall, the length of poly-LacNAc from G27Δ*HPG27_1230* LPS is similar to that of LPS from G27 wild-type.

As terminal Fuc and Gal were oxidised during the Smith degradation, we used a previously described NMR technique to further characterise Le^x^ and Le^y^ epitopes of the LPS samples [6], and corroborating evidence was supplied by NMR spectroscopy of the G27Δ*HPG27_1230* LPS. Fuc substitution of the LPS was investigated by inspection of cross-peaks in the TOCSY NMR spectrum between the well-resolved H6 and H5 signals of Fuc monosaccharide residues (**S2A Fig**). Two cross-peaks H6 1.23 ppm to H5 4.28 ppm and H6 1.14 ppm to H5 4.81 ppm can be assigned to terminal Fuc residues attached to Gal C2 and terminal Fuc attached to GlcNAc C3 respectively by comparison with published data [39]. These are consistent with the presence of Le^x^ and Le^y^ antigens. A third cross-peak H6 1.15 ppm to H5 4.34 ppm can be tentatively assigned to the 3-linked internal Fuc, as it is the only Fuc H6/H5 cross-peak in the TOCSY NMR spectra of G27Δ*HP1283* LPS (**S2B Fig**) and G27Δ*HP1578* (**S2C Fig**), both of which lack the Lewis antigen motifs.

The MALDI-TOF spectrum of HF hydrolysed G27Δ*HP1578* mutant LPS (**S1B Fig**) shows a long cluster of MS peaks at m/z 3000.7, 3248.8, 3496.9, 3745.0, 3993.1, 4241.2, 4489.4, 4737.4, 4985.6, 5233.7, 5481.7, 5729.7, 5977.8, 6226.1, 6474.1, 6722.1, 6970.1, 7218.5 and 7466.3, which are annotated as heptan-Glc5-Hep-Fuc structures containing Hep repeating for 6 to 24 times. The higher mass range (6000–7600 Da) of the spectrum is shown in **S1C Fig**. The MS peak at m/z 3000.7 was subjected to MS/MS analysis to confirm the linear heptan-glucan structure (**S1D Fig**). The MS/MS spectrum is divided by an ion at *m/z* 1497.6. The peak is assigned to a single-cleaved glycan fragment with a sequence of Glc5-Hep-Fuc based on smaller fragments at *m/z* 681.3, 885.3, 1089.3 and 1293.7 that carry different numbers of Glc units. A successive addition of a Hep fragment (248 Da) to the *m/z* 1497.6 peak gives rise to MS/MS peaks at *m/z* 1745.8, 1993.9 and 2242.1. These observations not only support a linear heptan-glucan architecture, but also confirm that the glucan contains 5 Glc repeating units. Importantly, the MS data indicate that G27Δ*HP1578* LPS carries a slightly longer heptan than the G27 wild-type LPS. We therefore propose HP1578 is the GlcNAc transferase that caps the heptan motif (**Fig 1C**).

Collectively, our systematic mutagenesis combined with LPS structural analysis suggest the identification of novel glycosyltransferase genes in G27 LPS biosynthesis: *HP0102*, encoding the Fuc transferase in the biosynthesis of the Trio structure; *HP1283*, encoding the heptan transferase, which is consistent with an earlier study [22], and *HP1578*, encoding the transferase which adds the GlcNAc residue to the heptan.

### Comparative genomic analysis of the complete G27 LPS gene set among *H*. *pylori* strains of different phylogeographic origins

**Fig 4:**
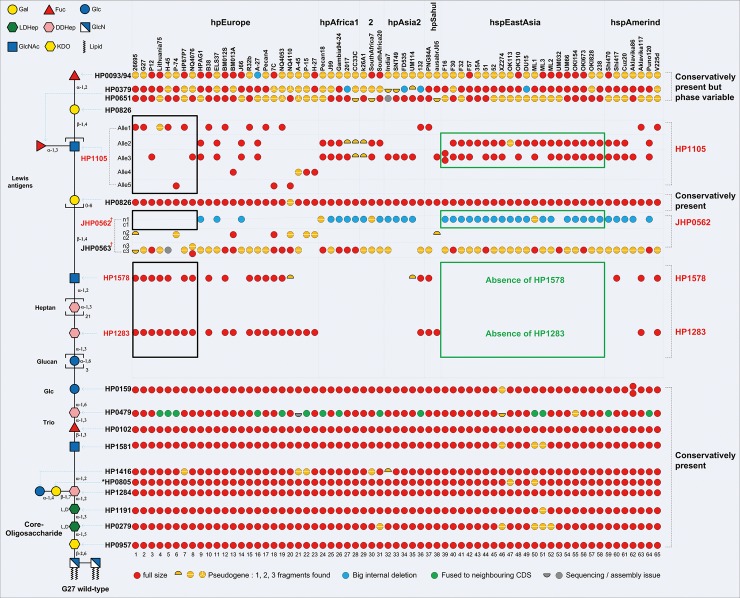
Bioinformatic analysis of the glycosyltransferase genes in *H*. *pylori* LPS biosynthetic pathway. The pattern of the presence/absence of the assigned LPS glycosyltransferase genes in 65 strains with well-assembled LPS genes. Based on the polymorphism of the carboxy-terminal half of the enzyme, 5 HP1105 alleles are distinguished. The locus *JHP0562-0563* contains one or two glycosyltransferase genes among the three possible ones (1 to 3) with the amino-terminal modules (n1 to n3) and the carboxy-terminal counterparts (c1 to c3). The glycosyltransferases responsible for synthesis of core-Trio-Glc and distal Lewis antigens are conserved amongst *H*. *pylori* strains; the glycosyltransferases responsible for synthesis of the intervening region between the core-Trio-Glc and the distal Lewis antigens vary substantially among *H*. *pylori* populations: both *HP1283* and *HP1578* are absent in all studied hspEastAsia strains exhibiting JHP0562 (n1c1) and two copies of HP1105 alleles (highlighted in green box). In contrast, strains harbouring the *HP1283/HP1578* usually contain only one copy of HP1105 (mostly allele 1) and lack *JHP0562* (n1c1) (highlighted in black box).

The above identification of the missing glycosyltransferase genes, together with confirmation of previously known LPS genes in the involvement of G27 LPS biosynthesis, enabled the complete assignment of LPS glycosyltransferase genes onto the corresponding G27 LPS structure (**Fig 4**, left schematic LPS structure). Of note, although the LPS from G27Δ*HP0805* was not subjected to structural analysis, HP0805 is postulated to transfer the Gal residue to the Hep III, based on the almost unaffected LPS length and Lewis antigen expression in G27Δ*HP0805* as compared to the G27 wild-type LPS (**Fig 2C**). Furthermore, HP0805, HP0826 and HP0619 are annotated as belonging to the same GT-25 family, and both HP0826 and JHP0563 (the functional HP0619) have been confirmed as Gal transferases [28,30]. Coupled with this information the glycosyltransferase genes *JHP0562* and *JHP0563*, though only present as non-functional fragments (*HPG27_579/580*) in the genome of G27, were also included for comparative genomic analysis.

With the complete G27 LPS glycosyltransferase gene set as a reference, a total of 177 genomes (including 132 public available *H*. *pylori* genomes at the time of this study, 44 newly-sequenced *H*. *pylori* isolates originating from our laboratory at West China hospital, and one Japanese strain CA2 with established LPS structures in a previous study [19]) were included for comparative genomic analysis (**S1 Table**). Multilocus sequence typing (MLST) analysis was performed using seven housekeeping genes. The included strains were classified into different populations: hpEurope (59), hpAfrica1 (15), hpAfrica2 (4), hpAsia2 (11), hpSahul (3), hspEastAsia (74) and hspAmerind (11) (**S1** and **S2 Tables**).

### Glycosyltransferase genes responsible for synthesizing the Core-Trio-Glc and the distal lewis antigens are conserved

Most of the 177 genomes of *H*. *pylori* strains were sequenced by Illumina, a second-generation sequencing technology producing very short reads which are not sufficiently long to allow the sequencing of repeated regions or gene segments found in several similar copies throughout a given genome. Thus, the long glycosyl transferases genes (*HP0379/HP0651*, *JHP0562/JHP0563*, and different *HP1105* alleles) which contain very similar sequences to each other, were not fully assembled in more than 100 of the *H*. *pylori* genomes in **S3 Table**. The comparative bioinformatic analysis in 65 of the genomes with well-assembled LPS genes, demonstrated that the glycosyltransferase genes involved in the biosynthesis of the core-oligosaccharide domain, the Trio, the glucan and the Lewis antigens were almost present in all the studied genomes (**Fig 4**). A detailed analysis revealed that the five glycosyltransferase genes (*HP0957*, *HP0279*, *HP1191*, *HP1284* and *HP1416*) involved in the biosynthesis of conserved core hexasaccharide (Glc-Gal-DD-Hep-LD-Hep-LD-Hep-KDO), the putative glycosyltransferase gene *HP0805*, the three glycosyltransferase genes (*wecA*, *HP0102* and *HP0479*) responsible for assembly of the Trio (Hep-Fuc-GlcNAc), the O-antigen ligase gene *waaL* and the Glc transferase gene *HP0159* are also highly conserved in the genome of all *H*. *pylori* strains examined (**Fig 4** and **S3 Table**).

The distribution of glycosyltransferase genes (*futA*, *fut*B, *futC*, *HP1105*, *Jhp0562/0563* and *HP0826*) known to be involved in the biosynthesis of Lewis antigens amongst the populations is rather complex (**Fig 4**). On the one hand, almost all of these biosynthetic genes (either intact or partial) are present in the genomes of all examined strains (**S3 Table**), providing supporting evidence at the genomic level that the potential to express Lewis antigens is a highly conserved feature of *H*. *pylori* LPS. On the other hand, most of these genes except *HP0826* are also subject to genetic mechanisms which most likely allow for the generation of additional diversity in the LPS structure. *HP0826*, the β-1,4-Gal transferase gene involved in the assembly of type 2 Lewis antigen LacNAc backbone GlcNAc-(β-1,4)-Gal is highly conserved, non-phase variable, and present in all studied strains (**Fig 4**).

Frameshift (F/S) within homopolymeric tracts (or sometimes dimer repeats) are commonly found in the three FucT genes *futA* (*HP0379*), *futB* (*HP0651*) and *futC* (*HP0093/94)* leading to the on/off switching nature of the genes (**S3 Table** red hashed lines) and consequent phase variation of Lewis antigen expression [32].

### The heptan transferase gene *HP1283* and the GlcNAc transferase gene *HP1578* are completely absent in East-Asian *H*. *pylori* Strains

The pattern of presence/absence of the heptan transferase gene *HP1283* and the GlcNAc transferase gene *HP1578* varies substantially among different *H*. *pylori* populations (**Fig 4** and **S3 Table**). The *HP1283* gene was observed to be frequently present in hpEurope (78%, 46/59) and hpSahul strains (100%, 3/3) (**S3 Table**). In addition, the *HP1283* was also found to be present in hpAfrica1 (2/15), hpAsia2 (4/11) and hspAmerind (2/11). Together, a total of 57 strains out of the studied 177 strains were identified to contain the *HP1283* gene (**S4 Table**), and the presence of *HP1578* was found to be associated with the presence of *HP1283* (**Fig 4** and **S3 Table**).

Intriguingly, the *HP1283/HP1578* genes were found to be completely absent in the 74 hspEastAsia strains, which was in sharp contrast to their common presence (78%) in the 59 hpEurope strains (**Fig 4**, **S3 Table**). It needs to be emphasized that at the commencement of the bioinformatics study, only the 30 East-Asian strains with public available genomes were included. Therefore, we undertook whole genome sequencing of 44 Chinese strains (prefixed with CHL-) which were later added to our bioinformatics analysis to confirm the absence of *HP1283* and *HP1578* genes in all East-Asian strains (**S1** and **S3 Tables**). Interestingly the two genes were also absent in the 4 available hpAfrica2 genomes, but at this stage more strains from this population need to be analysed to discover any correlations.

**Fig 5:**
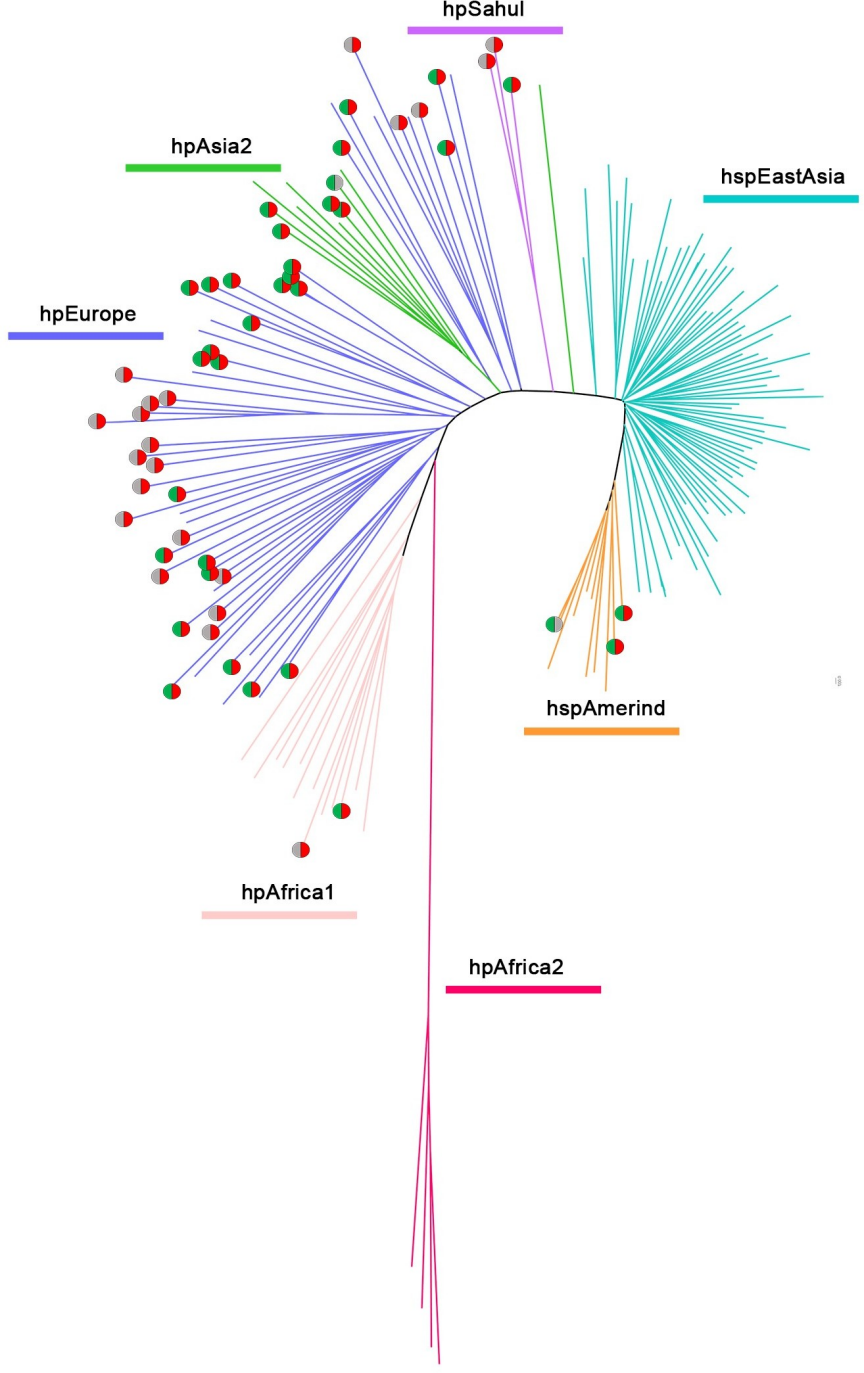
Phylogeographic distribution of the *HP1283/HP1578* genes in 177 *H*. *pylori* strains. The population and subpopulation of the 177 strains were assigned by population structure analysis based on Bayesian approach [49]. The presence of *HP1283* is coded by red, the presence of *HP1578* is coded green, whereas their absence is coded gray.

**Fig 6:**
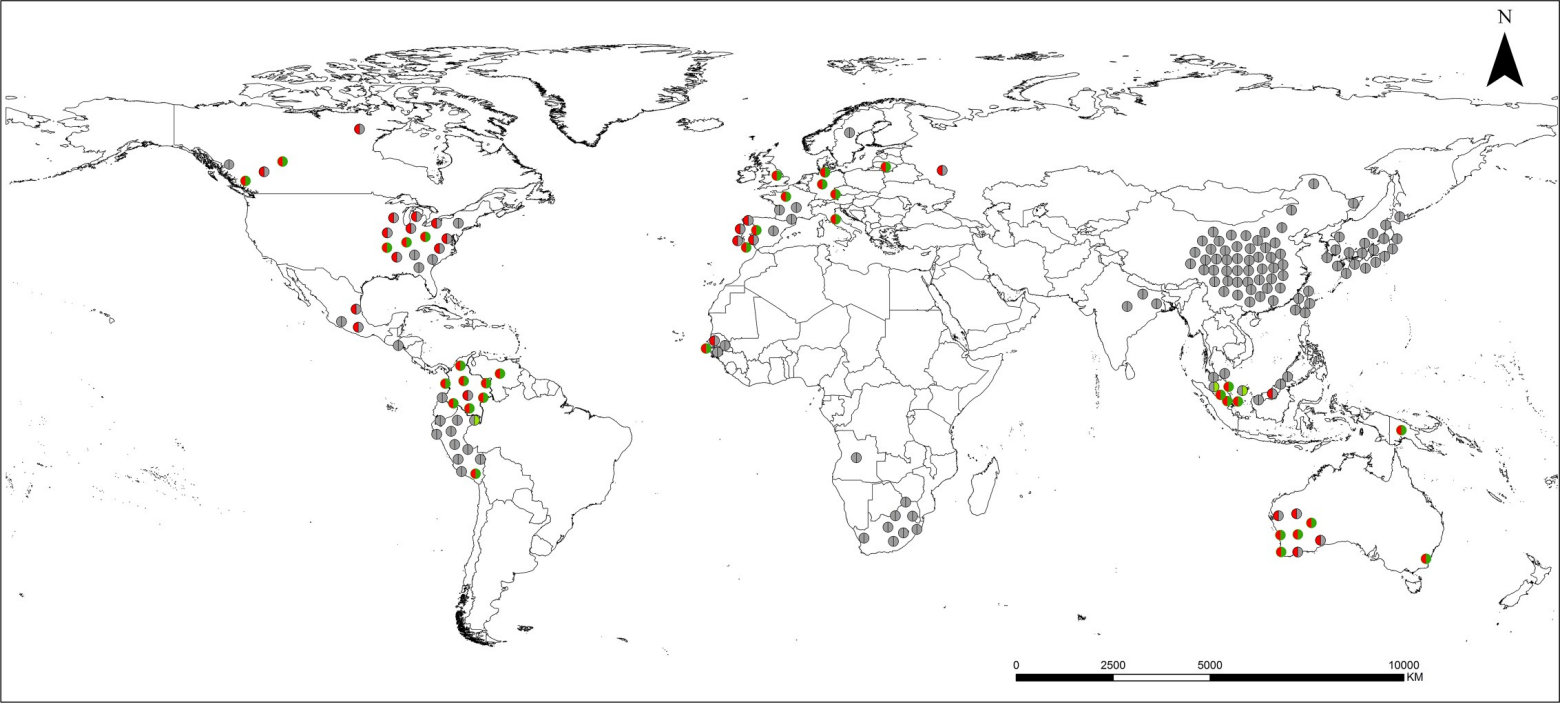
Geographic distribution of the *H*. *pylori* strains harbouring the *HP1283/1578* genes. The geographic origins of the 177 strains (S3 Table) were dot-graphed on the world map. The presence of *HP1283* is coded red, the presence of *HP1578* is coded green, whereas their absence is coded gray.

Collectively, the heptan transferase gene *HP1283* and the putative GlcNAc transferase gene *HP1578* are present in approximately 80% of Western *H*. *pylori* strains, whereas in East Asian strains there is a complete absence of these two genes (**Fig 5** and **Fig 6)**.

### Strains harbouring *HP1283/HP1578* contain only one copy of *HP1105* and no *JHP0562*, whereas strains lacking the *HP1283/HP1578* contain two copies of *HP1105* and *JHP0562*

The *HP1105* gene, coding a β-1,3-GlcNAc transferase, is present in at least one copy in all the studied strains but the peptide similarities can vary significantly from one strain to another (99% to less than 75%). It is possible to distinguish five different HP1105 alleles (**S3** and **S4 Figs**), based on the polymorphism of the carboxy-terminal half of the corresponding amino acid sequences, with the amino-terminal portion being highly conserved. A non-exhaustive summary of the HP1105 allele combinations in the examined strains is presented in **S3 Fig**. One copy of allele 1 (for which HP1105 in 26695 is the prototype) or allele 2 was found in less than 20% of the strains and apart from HPLT_05475 in Lithuania75, all the orthologs are assumed to be functional (**Fig 4**, **S3 Table**). Allele 1 and 2 seem more frequent in the hpEurope strains (including 26695 and G27). In contrast, these two alleles were not found in hspEastAsia and hpAfrica1/2 strains. More than 80% of the strains that do not harbour one of these two alleles bear, instead, at the same genetic locus, two copies of the three other more divergent paralogs (i.e. allele 3, 4 or 5). Strains containing two copies of this gene in tandem are frequent, with a majority of allele 3 and allele 4 (3+4) combinations. Other arrangements including tandem duplications (4+4 in strain F16) have also been observed but are much less frequent. None of the strains was found to harbour more than two full-size alleles simultaneously. Noteworthy, is that the recombinations responsible for these changes very rarely lead to hybrid glycosyltransferases, as was observed in the case of HPB8_399 (strain B8), which results from the (in frame) fusion of the *N*-terminal half of allele 3 with the c-terminal half of allele 4. Of note, the presence of two HP1105 alleles correlates with the absence of HP1283, except for three strains that contain both two HP1105 alleles and HP1283 (P-30, H-43 and A-27). Most strains with a single HP1105 allele (allele 1) harbour *HP1283* (**Fig 4, S3 Table**).

The *JHP0562* and *JHP0563* genes are also involved in Lewis antigen synthesis, and intragenomic recombination at this locus was proposed to generate diversity in Lewis antigens [27–29]. Depending on the strain, the *JHP0562/0563* locus in J99 (*HP0619* in 26695, *HPG27_579/580* in G27) contains one or two glycosyltransferase genes among the three possible ones (1 to 3, with an average size of 330, 440, and 400 amino acids, respectively). No strains were found to harbour all three genes simultaneously. The respective amino-terminal modules of the three possible glycosyltransferases (n1 to n3) differ sufficiently to be clearly distinguished from each other, and the same observation goes to their respective carboxy-terminal counterparts (c1 to c3). Despite these divergent sequences, genetic rearrangements are numerous and appear as the source of a great diversity of gene combinations (at least 16 of them may be found among the 177 strains analysed, **S5 Fig**). In general, the associations between the cognate n and c partner modules (i.e. n1 with c1, n2 with c2, n3 with c3) are preserved and the integrity of the GTs is not affected. However, similarly to what was observed with HP1105, true hybrid GTs (i.e with non-cognate n and c domains) may be detected. As exemplified by the cases of HP0619 in 26695 and HPG27_579/580 in G27 most of them are inactive because recombination resulted in a F/S between n and c modules. In strain J99, *JHP0562* represents the combination containing n1c1 without F/S and *JHP0563* is a combination of n3c3 with F/S (**S5 Fig**, combination 12). Of note, combination containing n1c1 without F/S like *JHP0562* in J99 (**S5 Fig**, combination 11, 12, 13 and 13d) were found more often in Asian strains and were usually exclusive of the presence *HP1283* gene (**Fig 4**, **S3 Table**). X568_03270 in SS1 (n2+c3) is a rare example of successful in frame fusion (**S3 Table**). The existence of F/S within a homopolymeric tract at the junction of modules n3 and c3 suggests that phase variation could be an additional diversity generator for this locus as reported before [27].

In summary, with rare exceptions, strains containing *HP1283/HP1578* harbour only one copy of *HP1105* and no *JHP0562*, whereas strains lacking the *HP1283/HP1578* contain two copies of *HP1105* and one copy of *JHP0562*.

## Discussion

The LPS of *H*. *pylori* plays essential roles in host-pathogen interactions, thus variations in *H*. *pylori* LPS structure and biosynthesis could substantially affect the pathological outcomes of host-pathogen interplay. This concept together with the international consensus classifying *H*. *pylori* as the most import risk factor of gastric cancer [2,3], with more than half coming from East Asia [1,4], prompted us to test our hypothesis that distinct differences in LPS gene content exist among *H*. *pylori* strains of different phylogeographic origin. Utilising bioinformatics, systematic mutagenesis of all known and putative LPS glycosyltranferase genes in a single G27 strain background, coupled with LPS structural studies by MS, we identified missing glycosytransferase genes underlying G27 LPS biosynthesis, leading to the establishment of the first complete LPS glycosyltransferase gene set in G27. Subsequently, using the complete G27 LPS gene set as a reference, comparative genomic analysis among *H*. *pylori* strains of different phylogeographic origin revealed the complete absence of the heptan transferase gene *HP1283*, and the newly identified GlcNAc transferase gene *HP1578* in East-Asian strains. This is consistent with the absence of the heptan moiety in established LPS structures from 12 East-Asian strains [19]. While the common occurrence of the LPS heptan moiety in Western *H*. *pylori* strains can now be explained by the common presence of the *HP1283/HP1578* genes in their genomes.

Prior to this study, several glycosyltransferase genes underlying G27 LPS biosynthesis remained unknown (**Fig 1A**). Here, a systematic deletion of 20 LPS genes in G27 enabled a thorough characterisation of the *H*. *pylori* LPS core-oligosaccharide and O-antigen biosynthetic pathway. LPS structural analysis of wild-type and isogenic mutants led to the assignment of *HP0102* as the Trio Fuc transferase gene; *HP1283* as the heptan transferase gene, which confirms recent work [22], and *HP1578* as the transferase gene responsible for adding the GlcNAc residue onto the heptan (**Fig 1**). Although the deletion of *HPG27_1230* in G27 led to a slight change to the LPS profile and the loss of Le^x^ on SDS-PAGE, the MS data indicated a similar LPS structure between G27Δ*HPG27_1230* and G27 wild-type. HPG27_1230 shares 41% protein sequence identity to HP1283, suggesting that HPG27_1230 is a HP1283-like protein. However, whether HPG27_1230 functions as a Hep transferase like HP1283 remains to be determined. *HP0805* is inferred to encode the transferase adding the Gal residue to the Hep III although structural analysis confirmation of LPS from Δ*HP0805* mutant is still required.

Our group has recently redefined the *H*. *pylori* LPS core-oligosaccharide as a short and highly conserved hexasaccharide, which in G27 is decorated with a long O-antigen encompassing the Trio, the intervening glucan-heptan, and the distal Lewis antigens [6]. This finding challenges the previous *H*. *pylori* LPS structural model in which the core-oligosacchride was divided into an inner and outer core and the O-antigen was composed exclusively of the Lewis antigens. In this study, the LPS length in mutants G27Δ*wecA*, G27Δ*wzk* and G27Δ*waaL* lacking the whole O-antigen was more severely truncated than that of mutants G27Δ*HP0826* and G27Δ*HP1105* lacking only the Lewis antigens (**Fig 2A and 2B**), providing further evidence to support our redefinition of *H*. *pylori* O-antigen as encompassing more than just the Lewis antigens. The observed successive truncation of the LPS demonstrated by each glycosyl transferase mutation (**Fig 2A and 2B**), together with structural validation of newly characterised *H*. *pylori* LPS mutants support a linear organization of G27 O-antigen domain with Lewis antigen at the tip, followed by heptan, glucan and Trio attached to the core oligosaccharide.

**Fig 7:**
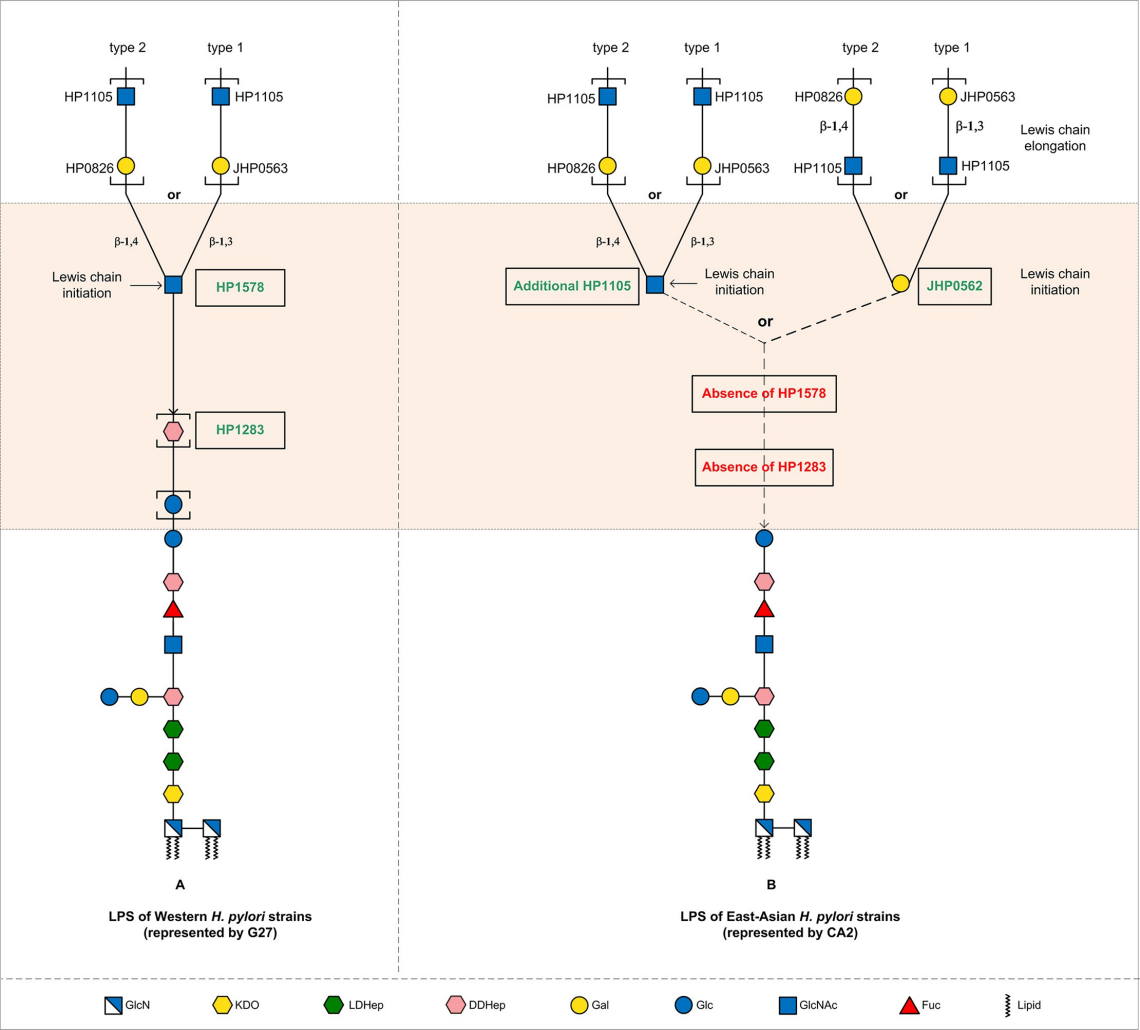
LPS biosynthesis model in *H*. *pylori*. The comparative genomic analysis of the LPS glycosyltransferase genes among *H*. *pylori* strains of different ethnic origin, provided genetic evidence for the structural conservation of the Glc-Trio-Core moiety of LPS, and the potential to express Lewis antigens in all *H*. *pylori* strains (Fig 4, S3 Table). However, the intermediate region (shaded) varies considerably between LPS from different *H*. *pylori* strains. (**A**): in Western strains (represented by G27) that harbour the heptan transferase gene *HP1283* and the GlcNAc transferase gene *HP1578*, the Lewis antigen synthesis is initiated by the GlcNAc transferase HP1578 onto the intermediate heptan (synthesized by HP1283); (**B**): in hspEAsia strains represented by the Japanese strains CA2 with its genome sequenced in this study showing the absence of *HP1283*/*HP1578* but the presence of two copies of *HP1105* and one copy of *JHP0562* (S3 Table column DS), and the established CA2 LPS structures lacking the intermediate heptan as reported in a previous study [19], the Lewis antigen is proposed to be directly attached onto the conserved Glc-Trio-Core structure via a GlcNAc or a Gal residue transferred by the additional HP1105 or JHP0562, respectively.

The comparative genomic analysis of the G27 LPS glycosyltransferase genes set in 177 diverse *H*. *pylori* strains, provided genetic evidence for the structural conservation of the Trio-Core moiety of LPS in all *H*. *pylori* strains examined (**Fig 4**, **S3 Table**). The gene *HP0159* encoding the transferase adding Glc residues after the Trio is also conserved. Interestingly, although Lewis antigen expression is known to be phase-variable, the genetic potential to express Lewis antigens seems to be highly conserved in *H*. *pylori* as well. In contrast, *HP1283* which encodes the heptan transferase underlying heptan biosynthesis was found to be completely absent in all hspEastAsia strains analysed in this study. This result suggests that the LPS in these strains does not contain heptan and is consistent with the lack of heptan reported in LPS from 12 strains isolated from China, Japan and Singapore [19]. Very interestingly, the absence of *HP1283* correlated with the absence of the newly discovered *HP1578* gene, in all hspEastAsia strains. Of note, we showed that in G27 *HP1283* and *HP1578* genes are required for the heptan biosynthesis and the GlcNAc transfer onto the heptan, respectively, enabling the successful initiation of Lewis antigen synthesis (**Fig 7A**). This raises the questions of how hspEastAsia strains, missing the heptan moiety, attach Lewis antigens onto the conserved Glc-Trio-Core.

In this regard, we looked for further correlation of LPS gene content related to the absence of genes *HP1283*/*HP1578* and uncovered that hspEastAsia strains, lacking *HP1283*/*HP1578*, usually harbour two *HP1105* alleles, compared to only the single *HP1105* allele found in most of the *H*. *pylori* strains harbouring *HP1283*/*HP1578* (**Fig 4** and **S3 Table**). Furthermore, the majority of these hspEastAsia strains contain the *JHP0562* allele, which is absent in nearly all strains with *HP1283*/*HP1578* (**S3 Table**). This suggests that in the absence of *HP1283*/*HP1578*, the additional *HP1105* and the *JHP0562* allele in these hspEastAsia strains might be crucial for attaching the Lewis antigens onto the conserved Glc-Trio-Core. It has been shown that the two *HP1105* alleles, *JHP1031* and *JHP1032* in J99 displaying 64% and 73% protein sequence identity to the *HP1105* in 26695, respectively [26]. Coupled enzymatic assays with JHP1032 and the β-1,4-Gal transferase HP0826 have been shown to be capable of synthesizing a tri-LacNAc product *in vitro*, demonstrating the β-1,3-GlcNAc transferase activity of JHP1032, which is the function of HP1105 in 26695 and G27, adding GlcNAc to Gal for the LacNAc backbone elongation [26]. Of note, in LPS synthesis of G27 and 26695, the single HP1105 allele is responsible for the LacNAc elongation [26], whereas the LacNAc initiation is conducted by the newly identified *HP1578*, encoding an α-1,2-GlcNAc transferase adding a GlcNAc to the heptan. The assignment of HP1578, HP1105 and JHP1031/1032 into the same GT8 family, and their homology at the amino acid level (**S6 Fig**) leads us to propose that the Lewis antigen, in hspEastAsia strains lacking heptan, can be initiated by the additional HP1105 transferring a GlcNAc onto the Glc-Trio-Core (**Fig 7B**, left arm). The LPS biosynthesis model in East-Asian strains is represented by the Japanese strain CA2 with its genome sequenced in this study showing the absence of *HP1283*/*HP1578* but the presence of two copies of *HP1105* and one copy of *JHP0562* (**S3 Table**, column DS), and its established LPS structures lacking the intermediate heptan reported in a previous study [19].

As to the role of *JHP0562*, Martin J. Blaser’s group has shown that it encodes a glycosyltransferase that it is required for the assembly of both type 1 and type 2 Lewis antigens [28]. JHP0562 shares a high degree of homology of with JHP0563 (the β-1,3-Gal transferase for adding a Gal to GlcNAc for type 1 Lewis chain elongation) and with HP0826 (the β-1,4-Gal transferase for adding a Gal to GlcNAc for type 2 Lewis chain elongation). As HP0826 and JHP0563 are involved in the elongation of type 2 and type 1 Lewis antigen backbone chain (Gal-β-1,4/3-GlcNAc), respectively, we propose that *JHP0562* may encode the Gal transferase responsible for the initiation of the assembly of both type 1 and type 2 Lewis antigens onto the Glc-Trio-Core (**Fig 7B**, right arm). This proposal would better explain the observation that the mutagenesis of *JHP0562* led to the abrogation of expression of both type 1 and type 2 Lewis antigen [28]. The role of *JHP0562* as a type 2 Lewis antigen initiating enzyme would also explain the observation that type 2 Le^y^ expression was not detected in the parent UM32 strain lacking a native *JHP0562*, whereas the acquisition of *JHP0562* led to the Le^y^ expression [29]. The established LPS structure in the mouse-adapted strain SS1 with a Gal residue directly attached onto the Glc-Trio [40], is also consistent with the presence of *JHP0562* in the SS1 genome (**S3 Table**), which encodes the corresponding Gal transferase to attach the Gal onto the Glc-Trio.

To summarise, we propose a *H*. *pylori* LPS biosynthetic model in which Lewis antigen biosynthesis can be initiated either by a GlcNAc (transferred by HP1578 or the additional HP1105) or a Gal residue (transferred by JHP0562) onto different acceptors with or without a heptan linker (transferred by HP1283) (**Fig 7**). Based on this model, the combination of the four LPS biosynthetic genes (*HP1283*, *HP1578*, *HP1105* and *JHP0562*) could reflect LPS structural differences in strains from diverse ethnic origins.

Finally, our data show geographic exclusion in East Asia of the presence of *HP1283* and *HP1578* genes in *H*. *pylori* strains (**Fig 6**). This observation raises the question of whether the *HP1283/HP1578* genes were lost in East Asian strains or acquired in European strains during human migration out of Africa [41]. Considering the recent discovery of the ADP-LD/DD-Hep (the precursor of the Hep residues present in *H*. *pylori* LPS core-oligosaccharide, Trio and DD-heptan) as a novel PAMP, which in *H*. *pylori* is CagT4SS-dependent to instigate the ALPK1-TIFA axis-mediated inflammatory response [13–15], it is tempting to postulate that the complete absence of the DD-heptan in East-Asian strains could affect the amount of the ADP-Hep delivered to the host cytosol by CagT4SS, thus the implication of the DD-heptan absence in gastric carcinogenesis. Additionally, the presence/absence of the heptan moiety in LPS structure might be involved in *H*. *pylori* pathogenesis as the presence of the heptan has been suggested to serve as a biological arm to facilitate the presentation of the Lewis antigens for host mimicry and immune escape [42].

## Materials and methods

### Bacterial strains, samples, culture and whole-genome sequencing

The G27 wild-type and its isogenic LPS mutants, plasmids, and oligonucleotides used in this study are listed in **S5** and **S6 Tables,** respectively. *H*. *pylori* strains were cultured as previously described [6].

The forty-four Chinese *H*. *pylori* isolates originated from patients belonging to the Han ethnic group. The lyophilized cells of the Japanese strain CA2 with a solved LPS structure [19], were kindly provided by Professor Shin-ichi Yokota (Department of Microbiology, Sapporo Medical University School of Medicine). Genomic DNA isolated from the above 45 East-Asian *H*. *pylori* strains using the QIAamp DNA Mini Kit (Qiagen), was subjected to whole-genome sequencing using an Illumina HiSeq X10 platform at Shenzhen BGI Diagnosis Technology Co., Ltd. The generated reads were decontaminated for any remaining illumina adapters using BBDuk program from BBtools suite (www.jgi.doe.gov/data-and-tools/bbtools/). The *de novo* assembly of reads was performed using SPAdes genome assembler (version 3.11.1) [43] and contigs of length less than 500 bp and coverage of 10 were removed. The sequences were then annotated using Prokka (Ver. 1.12) [44]. Draft genome sequences of the 44 Chinese strains and the Japanese strain CA2 were deposited at Genbank (**S1 Text**).

### Ethics statement

Gastroendoscopy was performed by two gastroenterologists (Y.X. and R.W.H.) with written informed consent at West China Hospital under ethics certificate 2017/332 approved by the Biomedical Research Ethics Committee.

### Systematic construction of LPS mutants and complementation

The deletion of *HP0805*, *HP0102* and *HP1283* in G27 was performed as previously described [45]. Other mutants were constructed using Xer-cise method [37]. Genetic complementation was performed using plasmid conjugation in a tri-parental mating format [46] **(S1 Text)**.

### LPS crude preparation for silver staining and western blot

LPS crude preparations from *H*. *pylori* wild-type and mutants were visualized on acrylamide gels by silver staining, and the presence of Lewis antigens was assessed by Western blot using mouse Anti-Le^x^ (1:1500) and Anti-Le^y^ (1:1500) as previously described [6].

### LPS structural analysis

LPS from G27Δ*HP0102*, G27Δ*HP1283*, G27Δ*HP1578* and G27Δ*HPG27_1230* was extracted and structurally analysed by mass spectrometry and NMR spectroscopy as previously described [6,47,48] **(S1 Text)**.

### Bioinformatic analysis

#### Data preparation

With the exception of the 45 newly sequenced strains in this study, the publicly available genomic data of the 132 *H*. *pylori* strains were retrieved from the NCBI genome page (www.ncbi.nlm.nih.gov/genome/). GenBank files containing single records or multi-record were preferentially used. Otherwise, original genome sequences were downloaded and annotated (i.e CDS prediction followed by automatic functional assignation and manual validation for the genes of interest).

#### Assignment of *H*. *pylori* population types

The SNPs extracted from the alignment of 7 housekeeping genes (*atpA*, *efp*, *mutY*, *ppa*, *trpC*, *ureI*, *yphC*) were subjected to STRUCTURE v.2.3.4 analysis [49], which implements a Bayesian approach to deduce the population structure. The Markov Chain Monte Carlo (MCMC) simulation underpinning STRUCTURE was run for 100,000 iterations, following a burn-in of 10,000 iterations, under the admixture model. The K in STRUCTURE was set to run from 4 to 12, with 10 repeats. Structure Harvester v0.6.94 [50], was then used to determine the optimal value of K. For sub-population identification, the same parameters were used on a smaller subset of strains.

#### Detection of LPS biosynthesis genes

CDS detection, annotation and comparison of LPS biosynthesis genes were carried out using M.A.G.D.A. (Multiple Annotation of Genomes and Differential Analysis, Center for Infection and Immunity of Lille, France), a bioinformatic tool optimized to facilitate the detection of phenotype-associated nucleotide or peptidic polymorphisms by simultaneously comparing up to several hundreds of genomes.

After automatic parsing of the genome files, an orthology matrix was constructed, based on the Bidirectional Best Hit (BDBH) results returned from tblastn queries. To avoid confusions between similar LPS biosynthesis genes and to detect eventual genome assembly issues or synteny breaks, analyses were systematically extended to the upstream and downstream flanking genes. Supporting tblastn results and alignments are available in the Supplementary Data File.

## Supporting information

S1 Fig MS and MS/MS Analysis G27 mutant LPS. (**A**): MALDI-TOF spectrum of G27Δ*HPG27_1230* LPS after Smith degradation; (**B**): MALDI-TOF spectrum of G27Δ*HP1578* LPS after mild HF hydrolysis; (**C**): MALDI-TOF spectrum of spectrum (B) zoomed into 6000–7600 Da mass range. Note spectrum (B) was annotated with theoretical mass-to-charge ratio, whereas spectrum (C) was annotated with observed average values. Red peaks corresponding to sodiated and permethylated glycans are annotated with mass-to-charge ratio and glycan structures; (**D)**: MALDI-TOF/TOF spectrum of the MS peak at m/z 3000.7 found in the spectrum (B). The MS data indicate G27Δ*HPG27_1230* LPS carries a longer profile of poly-lacNAc, and G27 Δ*HP1578* mutant LPS carries a longer heptan.
(TIF)

S2 Fig NMR TOCSY spectra of G27 mutant LPS. (**A**): G27ΔHPG27_1230 LPS; (**B**): G27ΔHP1283 LPS; (**C**): G27ΔHP1578 LPS, showing positive contours only. The LPS was incorporated into DPC micelles prior to the NMR experiments. The NMR spectrum was recorded by using a Bruker Avance III 600MHz NMR spectrometer equipped with a TXI/TCI cryoprobe. The spectra are zoomed into the region of proton H5-H6 cross-peaks of Fuc residues. Assignments marked in the figure are based on previously published Fuc chemical shifts [39].
(TIF)

S3 Fig HP1105 alignments. Five different HP1105 alleles can be distinguished in the *H*. *pylori* population based primarily on the polymorphism of the carboxy-terminal half of the HP1105 polypeptide sequences. Individual representatives of the five alleles: polypeptide sequences of HP1105 (allele 1) from strain 26695, HPHPP74_0722 (allele 2) from strain P-74, JHP_1031 (allele 3) and JHP_1032 (allele 4) from strain J99, EG63_05310 (allele 5) from strain BM013A were aligned.
(TIF)

S4 Fig Diverse HP1105 allele combination types among the examined H. pylori strains. Depending on the strain, the *HP1105* locus can be a single copy or different combinations of the same or different HP1105 alleles. Presented here is a non-exhaustive summary of the combination types found among the 176 strain analysed: 1–5, representative strains harbouring a single copy of the five different alleles, respectively; 4::3, strain B8 harbouring a hybrid allele resulting from fusion of the *N*-terminal half of allele 3 with the c-terminal half of allele 4; 4+2, strain P-30 harbouring simultaneously allele 2 and allele 4; 4+3, representative strains harbouring simultaneously allele 3 and allele 4; 4+4, strain F16 harbouring a tandem duplications of allele 4; 4+3Δ, strains 2017, 2018,wls-5-3 and 908 harbouring allele 4 and truncated allele 3; 4Δ+3Δ, representative strains harbouring both truncated allele 4 and truncated allele 3; 5Δ+4Δ+3Δ, strain NY40 harbouring truncated allele 5, truncated allele 4 and truncated allele 3.
(TIF)

S5 Fig Diversity of the JHP0562/0563 locus among the examined H. pylori strains categorised by combination types. Depending on the strain, the *jhp0562-0563* locus can be one or two glycosyltransferase genes among the three possible ones (1 to 3, with an average size of 330, 440, and 400 aminoacids respectively). The amino-terminal and carboxy-terminal modules of the three possible glycosyltransferases can be distinguished into n1-n3 and c1-c3, respectively. Genetic rearrangements of these different modules are numerous, and presented here is a non-exhaustive summary of the gene combinations found among the 176 strain analysed.
(TIF)

S6 Fig Alignments of HP1105, HP1578, JHP1031 and JHP1032 polypeptides. Alignments of polypeptide sequences of HP1105 and HP1578 from *H*. *pylori* strain 26695, and JHP1031 and JHP102 from strain J99 using MultAlin (http://bioinfo.genotoul.fr/multalin/multalin.html).
(TIF)

S1 Table Information of *H*. *pylori* strains included in this study. (DOCX)

S2 Table MSLT analysis of *H*. *pylori* strains. (XLSX)

S3 Table Comparative genomic analysis of LPS genes. (XLS)

S4 Table List of *H*. *pylori* strains containing HP1283. (DOCX)

S5 Table Plasmids and bacterial strains used in this study. (DOCX)

S6 Table Oligonucleotides used in this study. (DOCX)

S1 Text Detailed methods for the construction of LPS mutants and genetic complementation; detailed methods for the LPS structural analysis; and GenBank accession numbers for the 45 newly sequenced *H*. *pylori* genomes in this study. (DOCX)
