# Supplementary figures and images for: East-Asian Helicobacter pylori strains synthesize heptan-deficient lipopolysaccharide
Source: PLoS Genet. 2019 Nov 20;15(11):e1008497. doi: 10.1371/journal.pgen.1008497 (PMC6892558; doi:10.1371/journal.pgen.1008497)

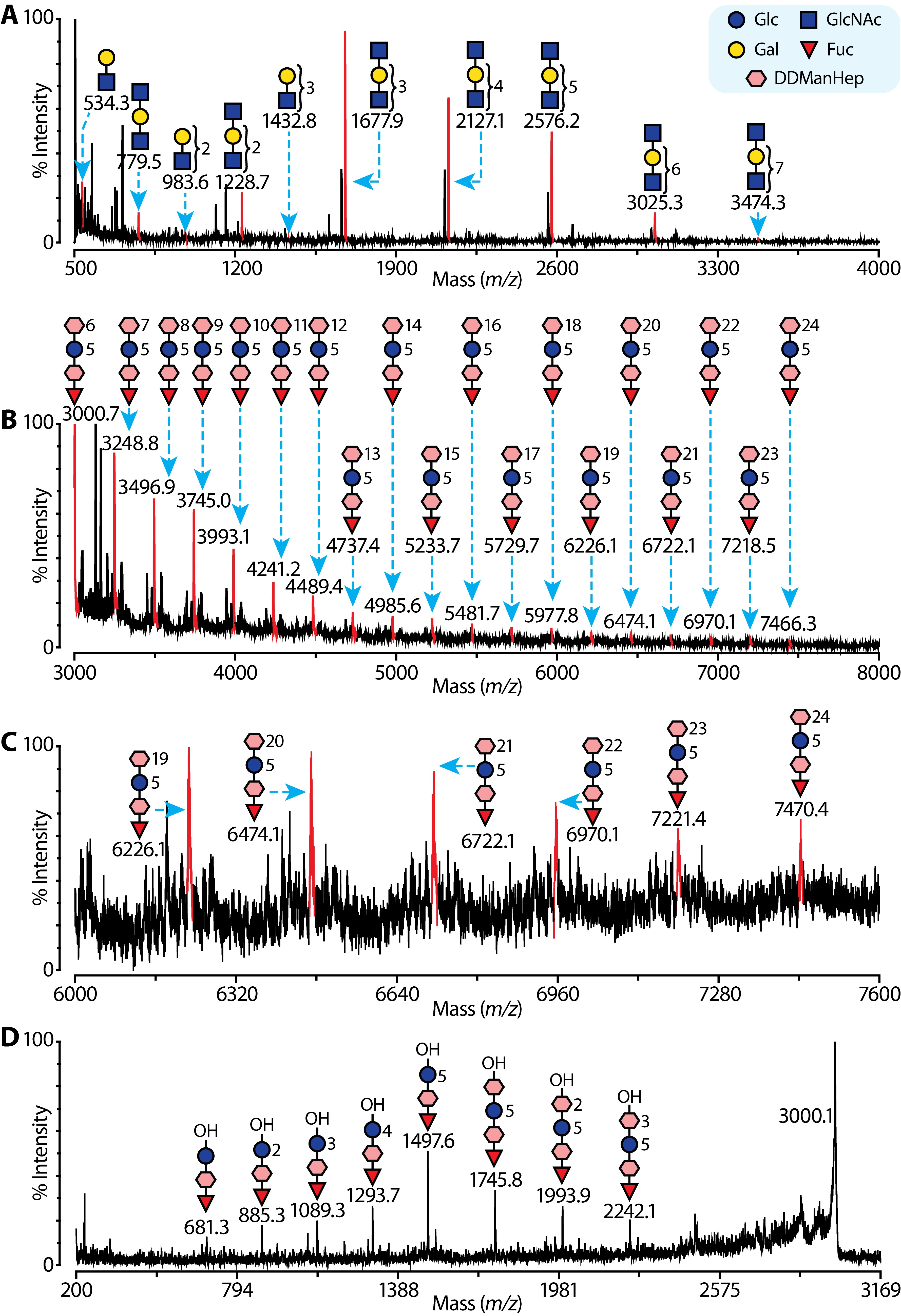

Supplement: S1 Fig — (A): MALDI-TOF spectrum of G27ΔHPG27_1230 LPS after Smith degradation; (B): MALDI-TOF spectrum of G27ΔHP1578 LPS after mild HF hydrolysis; (C): MALDI-TOF spectrum of spectrum (B) zoomed into 6000–7600 Da mass range. Note spectrum (B) was annotated with theoretical mass-to-charge ratio, whereas spectrum (C) was annotated with observed average values. Red peaks corresponding to sodiated and permethylated glycans are annotated with mass-to-charge ratio and glycan structures; (D): MALDI-TOF/TOF spectrum of the MS peak at m/z 3000.7 found in the spectrum (B). The MS data indicate G27ΔHPG27_1230 LPS carries a longer profile of poly-lacNAc, and G27 ΔHP1578 mutant LPS carries a longer heptan. (TIF) [file pgen.1008497.s001.tif]

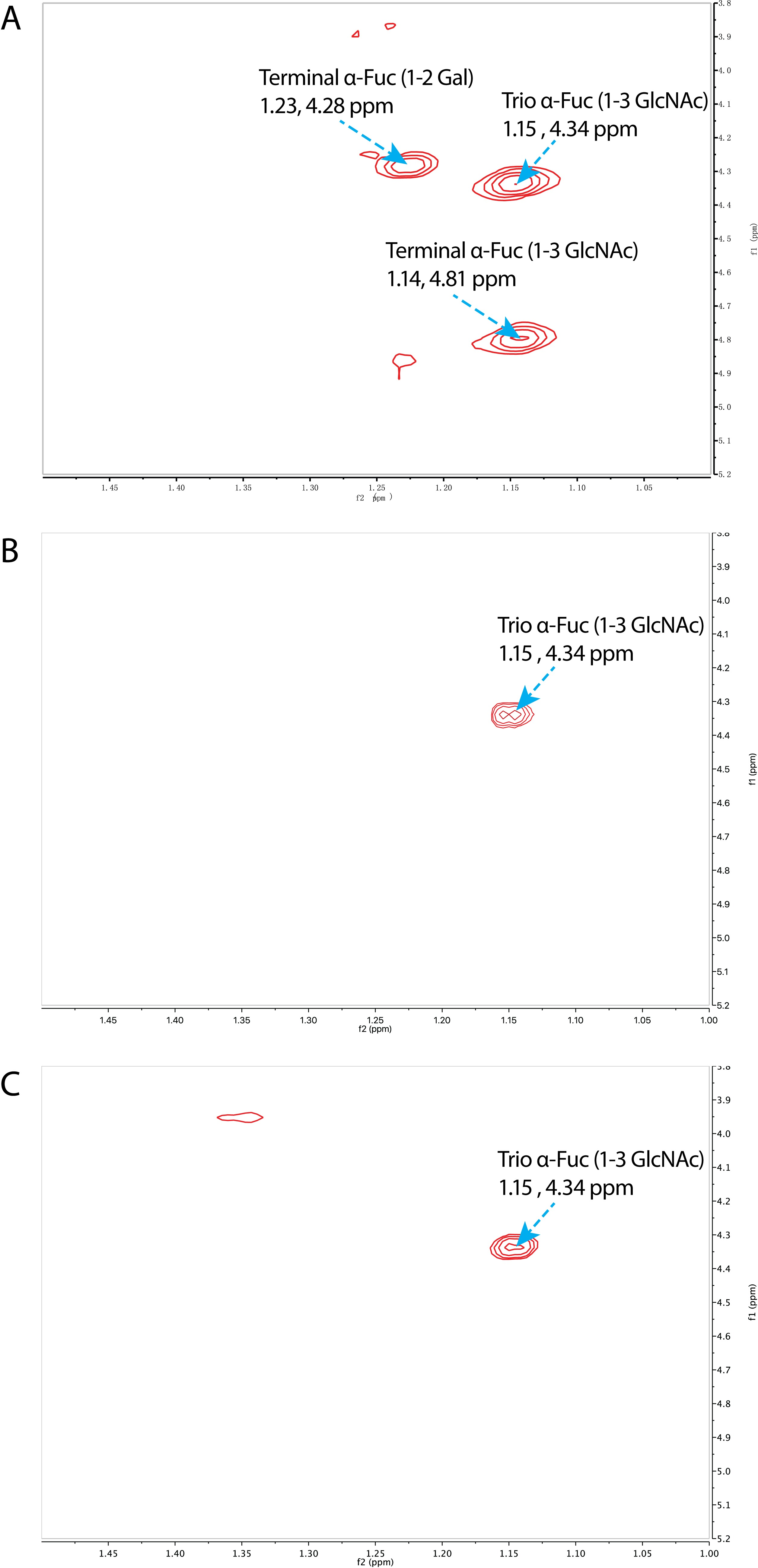

Supplement: S2 Fig — (A): G27ΔHPG27_1230 LPS; (B): G27ΔHP1283 LPS; (C): G27ΔHP1578 LPS, showing positive contours only. The LPS was incorporated into DPC micelles prior to the NMR experiments. The NMR spectrum was recorded by using a Bruker Avance III 600MHz NMR spectrometer equipped with a TXI/TCI cryoprobe. The spectra are zoomed into the region of proton H5-H6 cross-peaks of Fuc residues. Assignments marked in the figure are based on previously published Fuc chemical shifts [39]. (TIF) [file pgen.1008497.s002.tif]

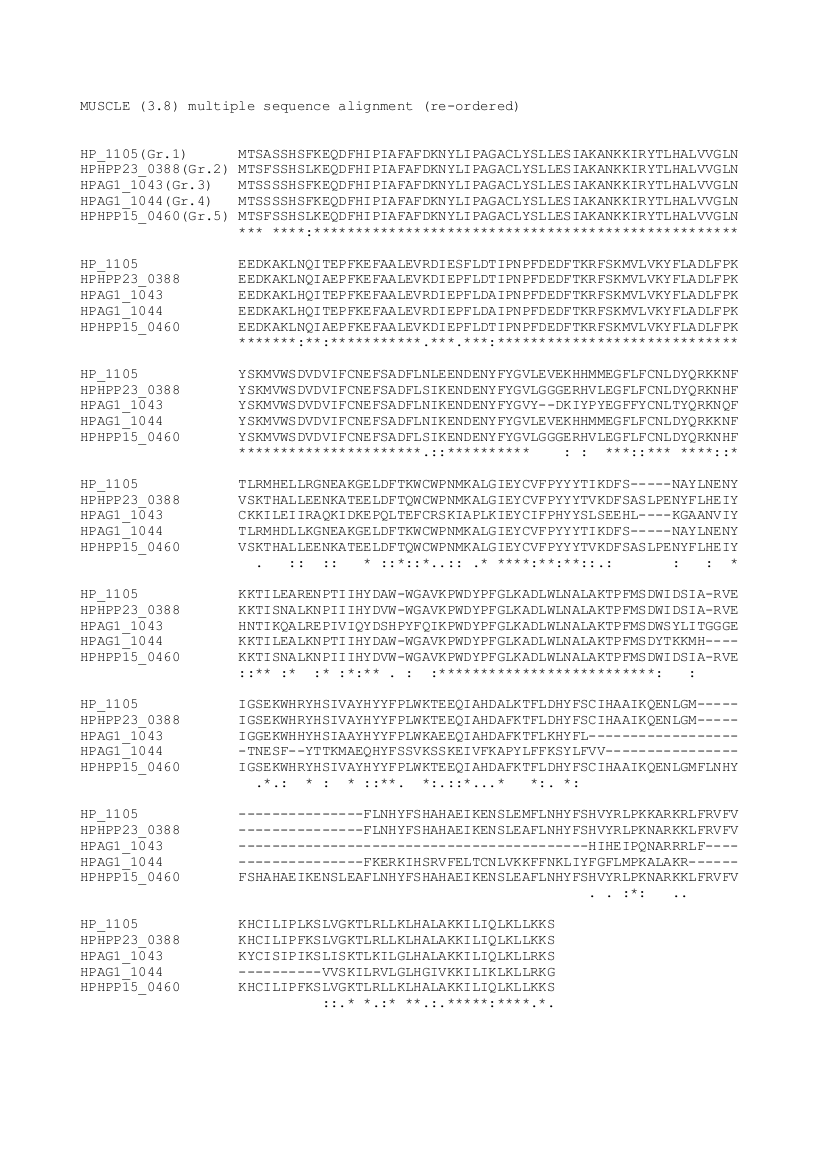

Supplement: S3 Fig — Five different HP1105 alleles can be distinguished in the H. pylori population based primarily on the polymorphism of the carboxy-terminal half of the HP1105 polypeptide sequences. Individual representatives of the five alleles: polypeptide sequences of HP1105 (allele 1) from strain 26695, HPHPP74_0722 (allele 2) from strain P-74, JHP_1031 (allele 3) and JHP_1032 (allele 4) from strain J99, EG63_05310 (allele 5) from strain BM013A were aligned. (TIF) [file pgen.1008497.s003.tif]

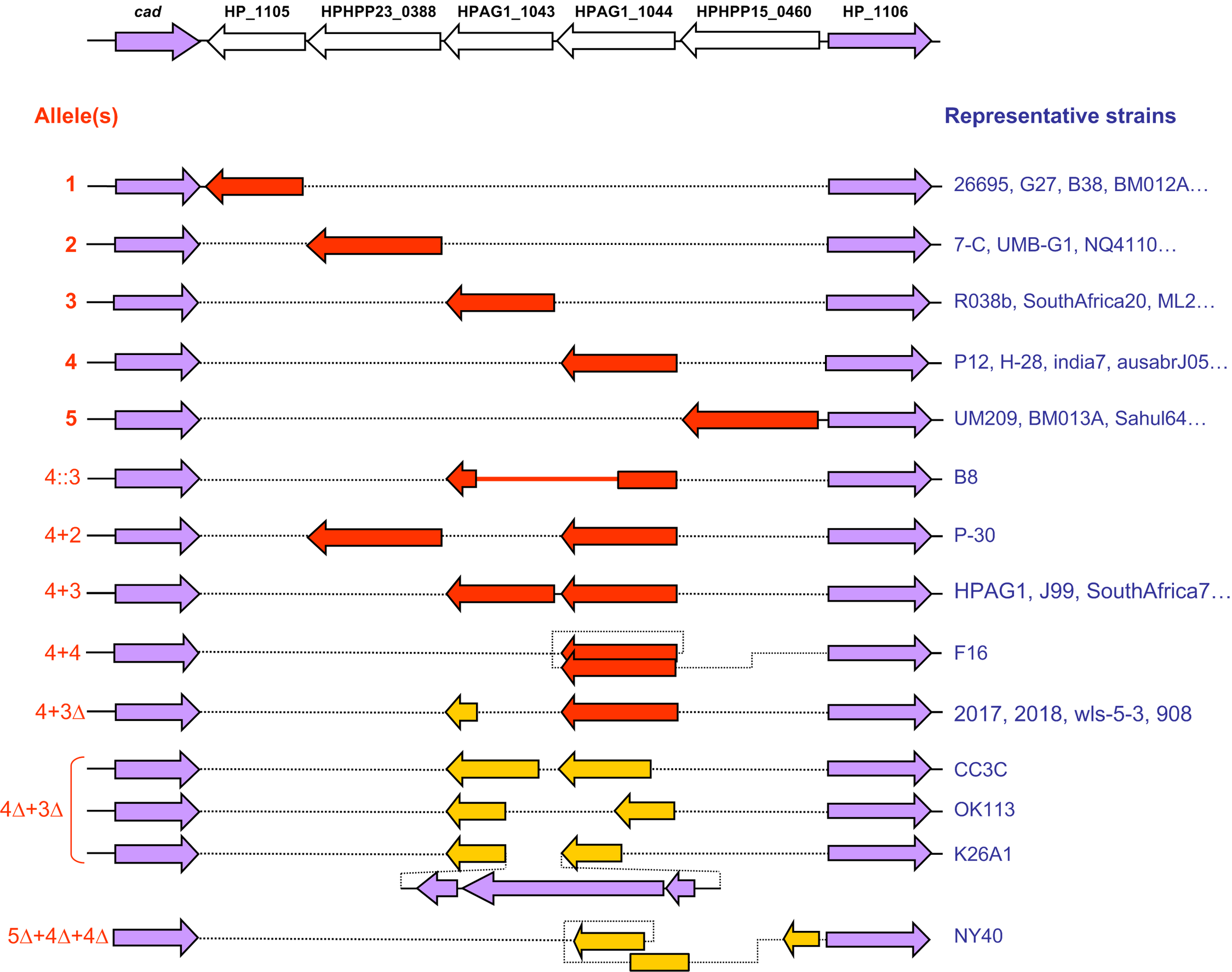

Supplement: S4 Fig — Depending on the strain, the HP1105 locus can be a single copy or different combinations of the same or different HP1105 alleles. Presented here is a non-exhaustive summary of the combination types found among the 176 strain analysed: 1–5, representative strains harbouring a single copy of the five different alleles, respectively; 4::3, strain B8 harbouring a hybrid allele resulting from fusion of the N-terminal half of allele 3 with the c-terminal half of allele 4; 4+2, strain P-30 harbouring simultaneously allele 2 and allele 4; 4+3, representative strains harbouring simultaneously allele 3 and allele 4; 4+4, strain F16 harbouring a tandem duplications of allele 4; 4+3Δ, strains 2017, 2018,wls-5-3 and 908 harbouring allele 4 and truncated allele 3; 4Δ+3Δ, representative strains harbouring both truncated allele 4 and truncated allele 3; 5Δ+4Δ+3Δ, strain NY40 harbouring truncated allele 5, truncated allele 4 and truncated allele 3. (TIF) [file pgen.1008497.s004.tif]

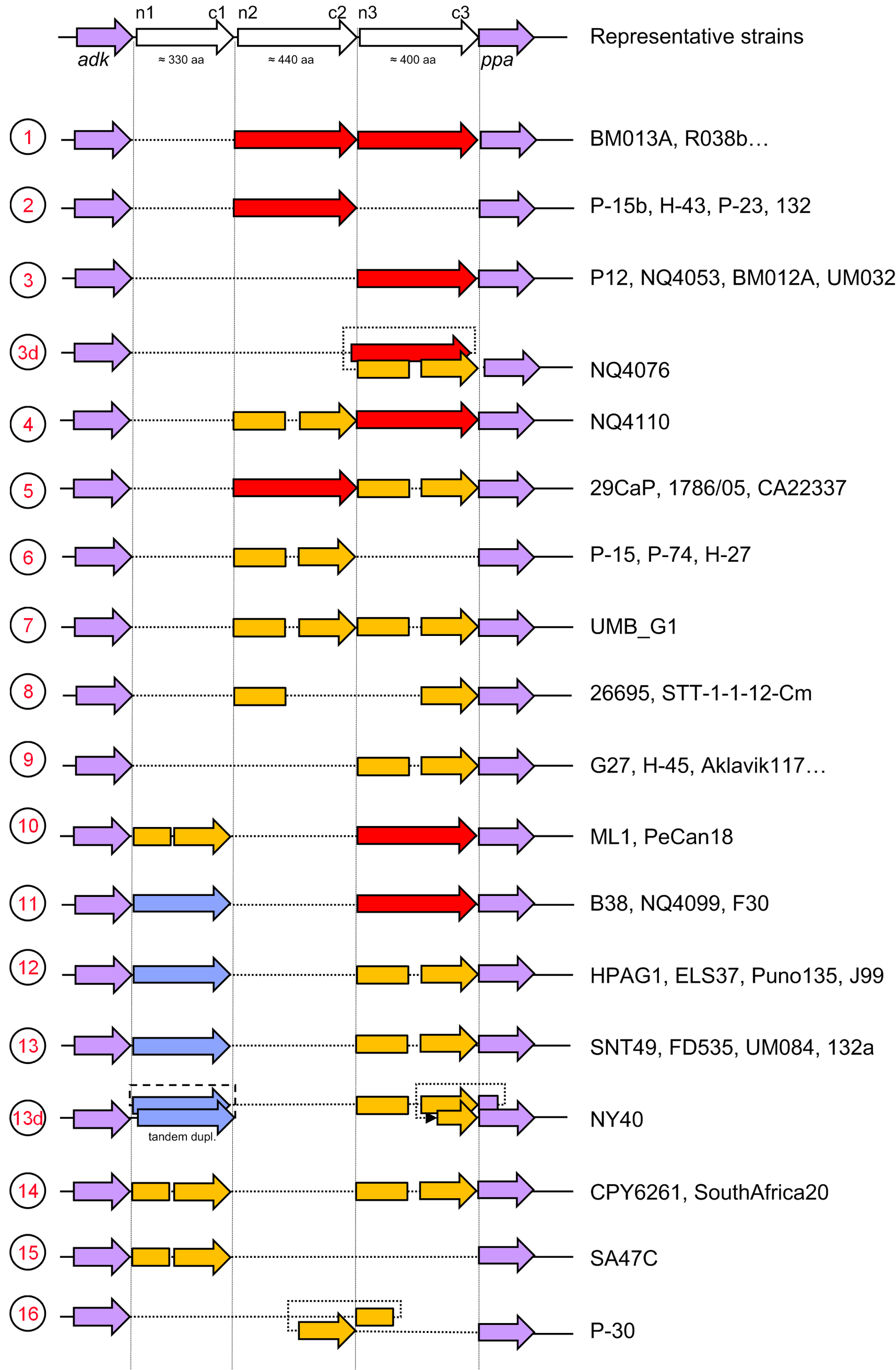

Supplement: S5 Fig — Depending on the strain, the jhp0562-0563 locus can be one or two glycosyltransferase genes among the three possible ones (1 to 3, with an average size of 330, 440, and 400 aminoacids respectively). The amino-terminal and carboxy-terminal modules of the three possible glycosyltransferases can be distinguished into n1-n3 and c1-c3, respectively. Genetic rearrangements of these different modules are numerous, and presented here is a non-exhaustive summary of the gene combinations found among the 176 strain analysed. (TIF) [file pgen.1008497.s005.tif]

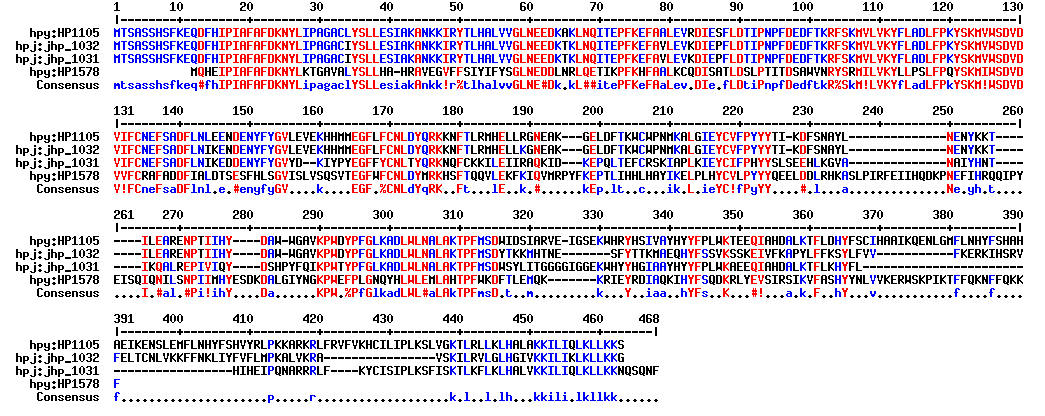

Supplement: S6 Fig — Alignments of polypeptide sequences of HP1105 and HP1578 from H. pylori strain 26695, and JHP1031 and JHP102 from strain J99 using MultAlin (http://bioinfo.genotoul.fr/multalin/multalin.html). (TIF) [file pgen.1008497.s006.tif]
