# Supplementary material for: East-Asian Helicobacter pylori strains synthesize heptan-deficient lipopolysaccharide
Source: PLoS Genet. 2019 Nov 20;15(11):e1008497. doi: 10.1371/journal.pgen.1008497 (PMC6892558; doi:10.1371/journal.pgen.1008497)
Supplement: S1 Table — (DOCX) [file pgen.1008497.s007.docx]

**S1 Table.** Information of *H. pylori* strains included in this study

| **NO.** | **Strain name** | **Geographical source** | **Isolation Country** | **Assigned population** | **References** |
| --- | --- | --- | --- | --- | --- |
|  | 26695 | Europe | UK | hpEurope | [[1](#_ENREF_1)] |
|  | G27 | Europe | Italy | hpEurope | [[2](#_ENREF_2)] |
|  | P12 | Europe | Germany | hpEurope | [[3](#_ENREF_3)] |
|  | Lithuania75 | Europe | Lithuania | hpEurope | - |
|  | 1786/05 | Europe | [Portugal](https://cn.bing.com/dict/search?q=Portugal&FORM=BDVSP6&mkt=zh-cn) | hpEurope | [[4](#_ENREF_4)] |
|  | Hp H-45 | North America | USA | hpEurope | [[5](#_ENREF_5)] |
|  | Hp P-23 | North America | USA | hpEurope | [[5](#_ENREF_5)] |
|  | Hp P-74 | North America | USA | hpEurope | [[5](#_ENREF_5)] |
|  | Hp H-28 | North America | USA | hpEurope | [[5](#_ENREF_5)] |
|  | HP87 P7 | Europe | Germany | hpEurope | [[6](#_ENREF_6)] |
|  | SS1 | Australia | Australia | hpEurope | [[7](#_ENREF_7)] |
|  | NQ4076 | South America | Colombia | hpEurope | [[5](#_ENREF_5)] |
|  | NQ4099 | South America | Colombia | hpEurope | [[5](#_ENREF_5)] |
|  | HUP-B14 | Europe | Spain | hpEurope | - |
|  | CHL-25 | East Asia | China | hpEurope | This study |
|  | 1846/05 | Europe | [Portugal](https://cn.bing.com/dict/search?q=Portugal&FORM=BDVSP6&mkt=zh-cn) | hpEurope | [[4](#_ENREF_4)] |
|  | STT-1-1-12-Cm | Europe | Germany | hpEurope | - |
|  | UM209 | Southeast Asia | Malaysia | hpEurope | - |
|  | NCTC11637 | Oceania | Australia | hpEurope | - |
|  | X47 | North America | USA | hpEurope | [[8](#_ENREF_8)] |
|  | HPAG1 | Europe | Sweden | hpEurope | [[9](#_ENREF_9)] |
|  | B8 | North America | USA | hpEurope | [[10](#_ENREF_10)] |
|  | B128 | North America | USA | hpEurope | [[11](#_ENREF_11)] |
|  | B38 | Europe | France | hpEurope | [[12](#_ENREF_12)] |
|  | ELS37 | Europe | El Salvador | hpEurope | - |
|  | SJM180 | South America | Peru | hpEurope | - |
|  | BM012A | Oceania | Australia | hpEurope | [[13](#_ENREF_13)] |
|  | BM012S | Oceania | Australia | hpEurope | [[13](#_ENREF_13)] |
|  | BM013A | Oceania | Australia | hpEurope | [[13](#_ENREF_13)] |
|  | BM013B | Oceania | Australia | hpEurope | [[13](#_ENREF_13)] |
|  | BM012B | Oceania | Australia | hpEurope | [[13](#_ENREF_13)] |
|  | J166 | North America | USA | hpEurope | [[13](#_ENREF_13)] |
|  | 207/99 | Europe | [Portugal](https://cn.bing.com/dict/search?q=Portugal&FORM=BDVSP6&mkt=zh-cn) | hpEurope | [[4](#_ENREF_4)] |
|  | 228//99 | Europe | [Portugal](https://cn.bing.com/dict/search?q=Portugal&FORM=BDVSP6&mkt=zh-cn) | hpEurope | [[4](#_ENREF_4)] |
|  | 655/99 | Europe | [Portugal](https://cn.bing.com/dict/search?q=Portugal&FORM=BDVSP6&mkt=zh-cn) | hpEurope | [[4](#_ENREF_4)] |
|  | 173/00 | Europe | [Portugal](https://cn.bing.com/dict/search?q=Portugal&FORM=BDVSP6&mkt=zh-cn) | hpEurope | [[4](#_ENREF_4)] |
|  | R32b | North America | Canada | hpEurope | [[5](#_ENREF_5)] |
|  | R038b | North America | Canada | hpEurope | [[5](#_ENREF_5)] |
|  | Hp P-30 | North America | USA | hpEurope | [[5](#_ENREF_5)] |
|  | Hp H-43 | North America | USA | hpEurope | [[5](#_ENREF_5)] |
|  | Hp A-27 | North America | USA | hpEurope | [[5](#_ENREF_5)] |
|  | Oki898 | East Asia | Japan | hpEurope | [[14](#_ENREF_14)] |
|  | Pecan4 | South America | Peru | hpEurope | - |
|  | 7C | North America | Mexico | hpEurope | [[15](#_ENREF_15)] |
|  | 29CaP | North America | Mexico | hpEurope | [[16](#_ENREF_16)] |
|  | UMB_G1 | North America | Canada | hpEurope | - |
|  | NQ4053 | South America | Colombia | hpEurope | [[5](#_ENREF_5)] |
|  | NQ4110 | South America | Colombia | hpEurope | [[5](#_ENREF_5)] |
|  | SV376_1 | South America | Colombia | hpEurope | - |
|  | UM037 | Southeast Asia | Malaysia | hpEurope | [[17](#_ENREF_17)] |
|  | NY40 | East Asia | Japan | hpEurope | - |
|  | CGIMSS2012 | N/A | N/A | hpEurope | - |
|  | CA22337 | South America | Colombia | hpEurope | [[18](#_ENREF_18)] |
|  | CA22362 | South America | Colombia | hpEurope | [[18](#_ENREF_18)] |
|  | CA26024 | South America | Colombia | hpEurope | [[18](#_ENREF_18)] |
|  | A45 | Europe | Russia | hpEurope | - |
|  | Hp P-15 | North America | USA | hpEurope | [[5](#_ENREF_5)] |
|  | Hp P-15b | North America | USA | hpEurope | [[5](#_ENREF_5)] |
|  | Hp H-27 | North America | USA | hpEurope | [[5](#_ENREF_5)] |
|  | PZ5033_3A2 | South America | Colombia | hpAfrica1 | - |
|  | PeCan18 | South America | Peru | hpAfrica1 | - |
|  | J99 | North America | US | hpAfrica1 | [[19](#_ENREF_19)] |
|  | Gambia94/24 | West Africa | Gambia | hpAfrica1 | - |
|  | GAM114Ai | West Africa | Gambia | hpAfrica1 | - |
|  | GAM264Ai | West Africa | Gambia | hpAfrica1 | - |
|  | 2017 | Europe | France | hpAfrica1 | [[20](#_ENREF_20)] |
|  | 2018 | Europe | France | hpAfrica1 | [[20](#_ENREF_20)] |
|  | 908 | Europe | France | hpAfrica1 | [[20](#_ENREF_20)] |
|  | SA162C | South Africa | South Africa | hpAfrica1 | [[21](#_ENREF_21)] |
|  | SA215C | South Africa | South Africa | hpAfrica1 | [[21](#_ENREF_21)] |
|  | SA220A | South Africa | South Africa | hpAfrica1 | [[21](#_ENREF_21)] |
|  | CC33C | South Africa | Capetown | hpAfrica1 | - |
|  | 250BFi | N/A | N/A | hpAfrica1 | - |
|  | Gambia201Ai | West Africa | Gambia | hpAfrica1 | - |
|  | K26A1 | South Africa | Angola | hpAfrica2 | - |
|  | SouthAfrica7 | South Africa | South Africa | hpAfrica2 | [[22](#_ENREF_22)] |
|  | SouthAfrica20 | South Africa | South Africa | hpAfrica2 | [[22](#_ENREF_22)] |
|  | SA47C | South Africa | South Africa | hpAfrica2 | [[21](#_ENREF_21)] |
|  | India7 | South Asia | India | hpAsia2 | - |
|  | SNT49 | South Asia | India | hpAsia2 | - |
|  | L7 | South Asia | India | hpAsia2 | - |
|  | FD535 | Southeast Asia | Malaysia (indian) | hpAsia2 | [[17](#_ENREF_17)] |
|  | FD703 | Southeast Asia | Malaysia (Malay) | hpAsia2 | [[17](#_ENREF_17)] |
|  | FD423 | Southeast Asia | Malaysia (indian) | hpAsia2 | [[17](#_ENREF_17)] |
|  | UM114 | Southeast Asia | Malaysia (indian) | hpAsia2 | [[17](#_ENREF_17)] |
|  | UM084 | Southeast Asia | Malaysia (Malay) | hpAsia2 | [[17](#_ENREF_17)] |
|  | 132 | Southeast Asia | Malaysia | hpAsia2 | - |
|  | 132A | Southeast Asia | Malaysia | hpAsia2 | - |
|  | UM087 | Southeast Asia | Malaysia | hpAsia2 | - |
|  | PNG84A | Oceania | Papua New Guinea | hpSahul | - |
|  | ausabrJ05 | Oceania | [Australia](https://www.ncbi.nlm.nih.gov/biosample?term=%22geo_loc_name=Jigalong,%20Australia%22%5battr%5d) | hpSahul | - |
|  | Sahul64 | Oceania | Australia | hpSahul | [[23](#_ENREF_23)] |
|  | F16 | East Asia | Japan | hspEastAsia | [[24](#_ENREF_24)] |
|  | F30 | East Asia | Japan | hspEastAsia | [[24](#_ENREF_24)] |
|  | F32 | East Asia | Japan | hspEastAsia | [[24](#_ENREF_24)] |
|  | F57 | East Asia | Japan | hspEastAsia | [[24](#_ENREF_24)] |
|  | 35A | East Asia | Japan | hspEastAsia | [[24](#_ENREF_24)] |
|  | 83 | East Asia | Japan | hspEastAsia | - |
|  | 51 | East Asia | South Korea | hspEastAsia | - |
|  | 52 | East Asia | South Korea | hspEastAsia | - |
|  | XZ274 | East Asia | China | hspEastAsia | [[25](#_ENREF_25)] |
|  | YN4-84 | East Asia | China | hspEastAsia | [[26](#_ENREF_26)] |
|  | YN1-91 | East Asia | China | hspEastAsia | [[26](#_ENREF_26)] |
|  | HLJHP193 | East Asia | China | hspEastAsia | - |
|  | HLJHP253 | East Asia | China | hspEastAsia | - |
|  | HLJHP256 | East Asia | China | hspEastAsia | - |
|  | OK113 | East Asia | Japan | hspEastAsia | [[27](#_ENREF_27)] |
|  | OK310 | East Asia | Japan | hspEastAsia | [[27](#_ENREF_27)] |
|  | CPY6311 | East Asia | Japan | hspEastAsia | [[5](#_ENREF_5)] |
|  | CPY6261 | East Asia | Japan | hspEastAsia | [[5](#_ENREF_5)] |
|  | DU15 | East Asia | South Korea | hspEastAsia | - |
|  | HPML1 | East Asia | China | hspEastAsia | [[28](#_ENREF_28)] |
|  | HPML2 | East Asia | China | hspEastAsia | [[28](#_ENREF_28)] |
|  | HPML3 | East Asia | China | hspEastAsia | [[28](#_ENREF_28)] |
|  | wls-5-3 | East Asia | China | hspEastAsia | - |
|  | 98-10 | East Asia | Japan | hspEastAsia | [[11](#_ENREF_11)] |
|  | UM032 | Southeast Asia | Malaysia (Chinese) | hspEastAsia | [[29](#_ENREF_29)] |
|  | UM066 | Southeast Asia | Malaysia (Chinese) | hspEastAsia | - |
|  | CA2 | East Asia | Japan | hspEastAsia | [[30](#_ENREF_30)] |
|  | CHL-01 | East Asia | China | hspEastAsia | This study |
|  | CHL-02 | East Asia | China | hspEastAsia | This study |
|  | CHL-03 | East Asia | China | hspEastAsia | This study |
|  | CHL-04 | East Asia | China | hspEastAsia | This study |
|  | CHL-05 | East Asia | China | hspEastAsia | This study |
|  | CHL-06 | East Asia | China | hspEastAsia | This study |
|  | CHL-07 | East Asia | China | hspEastAsia | This study |
|  | CHL-08 | East Asia | China | hspEastAsia | This study |
|  | CHL-09 | East Asia | China | hspEastAsia | This study |
|  | CHL-10 | East Asia | China | hspEastAsia | This study |
|  | CHL-11 | East Asia | China | hspEastAsia | This study |
|  | CHL-12 | East Asia | China | hspEastAsia | This study |
|  | CHL-14 | East Asia | China | hspEastAsia | This study |
|  | CHL-16 | East Asia | China | hspEastAsia | This study |
|  | CHL-17 | East Asia | China | hspEastAsia | This study |
|  | CHL-19 | East Asia | China | hspEastAsia | This study |
|  | CHL-20 | East Asia | China | hspEastAsia | This study |
|  | CHL-21 | East Asia | China | hspEastAsia | This study |
|  | CHL-22 | East Asia | China | hspEastAsia | This study |
|  | CHL-23 | East Asia | China | hspEastAsia | This study |
|  | CHL-24 | East Asia | China | hspEastAsia | This study |
|  | CHL-26 | East Asia | China | hspEastAsia | This study |
|  | CHL-27 | East Asia | China | hspEastAsia | This study |
|  | CHL-29 | East Asia | China | hspEastAsia | This study |
|  | CHL-31 | East Asia | China | hspEastAsia | This study |
|  | CHL-32 | East Asia | China | hspEastAsia | This study |
|  | CHL-33 | East Asia | China | hspEastAsia | This study |
|  | CHL-35 | East Asia | China | hspEastAsia | This study |
|  | CHL-36 | East Asia | China | hspEastAsia | This study |
|  | CHL-37 | East Asia | China | hspEastAsia | This study |
|  | CHL-38 | East Asia | China | hspEastAsia | This study |
|  | CHL-39 | East Asia | China | hspEastAsia | This study |
|  | CHL-41 | East Asia | China | hspEastAsia | This study |
|  | CHL-42 | East Asia | China | hspEastAsia | This study |
|  | CHL-44 | East Asia | China | hspEastAsia | This study |
|  | CHL-46 | East Asia | China | hspEastAsia | This study |
|  | CHL-47 | East Asia | China | hspEastAsia | This study |
|  | CHL-48 | East Asia | China | hspEastAsia | This study |
|  | CHL-49 | East Asia | China | hspEastAsia | This study |
|  | CHL-50 | East Asia | China | hspEastAsia | This study |
|  | CHL-51 | East Asia | China | hspEastAsia | This study |
|  | CHL-52 | East Asia | China | hspEastAsia | This study |
|  | CHL-54 | East Asia | China | hspEastAsia | This study |
|  | Oki154 | East Asia | Japan | hspEastAsia | [[14](#_ENREF_14)] |
|  | Oki673 | East Asia | Japan | hspEastAsia | [[14](#_ENREF_14)] |
|  | Oki828 | East Asia | Japan | hspEastAsia | [[14](#_ENREF_14)] |
|  | 238 | East Asia | China | hspEastAsia | - |
|  | Shi169 | South America | Peru | hspAmerind | - |
|  | Shi470 | South America | Peru | hspAmerind | - |
|  | Shi417 | South America | Peru | hspAmerind | - |
|  | Shi112 | South America | Peru | hspAmerind | - |
|  | Cuz20 | South America | Peru | hspAmerind | - |
|  | Aklavik86 | North America | Canada | hspAmerind | [[31](#_ENREF_31)] |
|  | Aklavik117 | North America | Canada | hspAmerind | [[31](#_ENREF_31)] |
|  | Puno120 | South America | Peru | hspAmerind | - |
|  | Sat464 | South America | Peru | hspAmerind | - |
|  | Puno135 | South America | Peru | hspAmerind | - |
|  | V225d | South America | Venezuela | hspAmerind | [[32](#_ENREF_32)] |

- Isolates with no publication associated to them

**References**

1. Tomb JF, White O, Kerlavage AR, Clayton RA, Sutton GG, et al. (1997) The complete genome sequence of the gastric pathogen Helicobacter pylori. Nature 388: 539-547.

2. Baltrus DA, Amieva MR, Covacci A, Lowe TM, Merrell DS, et al. (2009) The complete genome sequence of Helicobacter pylori strain G27. J Bacteriol 191: 447-448.

3. Fischer W, Windhager L, Rohrer S, Zeiller M, Karnholz A, et al. (2010) Strain-specific genes of Helicobacter pylori: genome evolution driven by a novel type IV secretion system and genomic island transfer. Nucleic Acids Res 38: 6089-6101.

4. Nunes A, Rocha R, Vale FF, Vieira L, Sampaio DA, et al. (2015) Genome Sequencing of 10 Helicobacter pylori Pediatric Strains from Patients with Nonulcer Dyspepsia and Peptic Ulcer Disease. 3.

5. Blanchard TG, Czinn SJ, Correa P, Nakazawa T, Keelan M, et al. (2013) Genome sequences of 65 Helicobacter pylori strains isolated from asymptomatic individuals and patients with gastric cancer, peptic ulcer disease, or gastritis. Pathog Dis 68: 39-43.

6. Behrens W, Schweinitzer T, Bal J, Dorsch M, Bleich A, et al. (2013) Role of energy sensor TlpD of Helicobacter pylori in gerbil colonization and genome analyses after adaptation in the gerbil. Infect Immun 81: 3534-3551.

7. Draper JL, Hansen LM, Bernick DL, Abedrabbo S, Underwood JG, et al. (2017) Fallacy of the Unique Genome: Sequence Diversity within Single Helicobacter pylori Strains. 8.

8. Handt LK, Fox JG, Stalis IH, Rufo R, Lee G, et al. (1995) CHARACTERIZATION OF FELINE HELICOBACTER-PYLORI STRAINS AND ASSOCIATED GASTRITIS IN A COLONY OF DOMESTIC CATS. Journal of Clinical Microbiology 33: 2280-2289.

9. Oh JD, Kling-Backhed H, Giannakis M, Xu J, Fulton RS, et al. (2006) The complete genome sequence of a chronic atrophic gastritis Helicobacter pylori strain: Evolution during disease progression. Proceedings of the National Academy of Sciences of the United States of America 103: 9999-10004.

10. Farnbacher M, Jahns T, Willrodt D, Daniel R, Haas R, et al. (2010) Sequencing, annotation, and comparative genome analysis of the gerbil-adapted Helicobacter pylori strain B8. BMC Genomics 11: 335.

11. McClain MS, Shaffer CL, Israel DA, Peek RM, Jr., Cover TL (2009) Genome sequence analysis of Helicobacter pylori strains associated with gastric ulceration and gastric cancer. BMC Genomics 10: 3.

12. Thiberge JM, Boursaux-Eude C, Lehours P, Dillies MA, Creno S, et al. (2010) From array-based hybridization of Helicobacter pylori isolates to the complete genome sequence of an isolate associated with MALT lymphoma. BMC Genomics 11: 368.

13. Linz B, Windsor HM, McGraw JJ, Hansen LM, Gajewski JP, et al. (2014) A mutation burst during the acute phase of Helicobacter pylori infection in humans and rhesus macaques. Nat Commun 5: 4165.

14. Satou K, Shiroma A, Teruya K, Shimoji M, Nakano K, et al. (2014) Complete Genome Sequences of Eight Helicobacter pylori Strains with Different Virulence Factor Genotypes and Methylation Profiles, Isolated from Patients with Diverse Gastrointestinal Diseases on Okinawa Island, Japan, Determined Using PacBio Single-Molecule Real-Time Technology. Genome Announc 2.

15. Mucito-Varela E, Castillo-Rojas G, Cevallos MA, Lozano L, Merino E, et al. (2016) Complete Genome Sequence of Helicobacter pylori Strain 7C Isolated from a Mexican Patient with Chronic Gastritis. 4.

16. Mucito-Varela E, Castillo-Rojas G, Cevallos MA, Lozano L, Merino E, et al. (2016) Complete Genome Sequence of Helicobacter pylori Strain 29CaP Isolated from a Mexican Patient with Gastric Cancer. 4.

17. Rehvathy V, Tan MH, Gunaletchumy SP, Teh X, Wang S, et al. (2013) Multiple Genome Sequences of Helicobacter pylori Strains of Diverse Disease and Antibiotic Resistance Backgrounds from Malaysia. Genome Announc 1.

18. Munoz-Ramirez ZY, Mendez-Tenorio A, Kato I, Bravo MM, Rizzato C, et al. (2017) Whole Genome Sequence and Phylogenetic Analysis Show Helicobacter pylori Strains from Latin America Have Followed a Unique Evolution Pathway. Front Cell Infect Microbiol 7: 50.

19. Alm RA, Ling LSL, Moir DT, King BL, Brown ED, et al. (1999) Genomic-sequence comparison of two unrelated isolates of the human gastric pathogen Helicobacter pylori. Nature 397: 719-719.

20. Avasthi TS, Devi SH, Taylor TD, Kumar N, Baddam R, et al. (2011) Genomes of two chronological isolates (Helicobacter pylori 2017 and 2018) of the West African Helicobacter pylori strain 908 obtained from a single patient. J Bacteriol 193: 3385-3386.

21. Didelot X, Nell S, Yang I, Woltemate S, van der Merwe S, et al. (2013) Genomic evolution and transmission of Helicobacter pylori in two South African families. Proc Natl Acad Sci U S A 110: 13880-13885.

22. Duncan SS, Bertoli MT, Kersulyte D, Valk PL, Tamma S, et al. (2013) Genome Sequences of Three hpAfrica2 Strains of Helicobacter pylori. Genome Announc 1.

23. Lu W, Wise MJ, Tay CY, Windsor HM, Marshall BJ, et al. (2014) Comparative analysis of the full genome of Helicobacter pylori isolate Sahul64 identifies genes of high divergence. J Bacteriol 196: 1073-1083.

24. Kawai M, Furuta Y, Yahara K, Tsuru T, Oshima K, et al. (2011) Evolution in an oncogenic bacterial species with extreme genome plasticity: Helicobacter pylori East Asian genomes. BMC Microbiol 11: 104.

25. Guo Y, Wang H, Li Y, Song Y, Chen C, et al. (2012) Genome of Helicobacter pylori strain XZ274, an isolate from a tibetan patient with gastric cancer in China. J Bacteriol 194: 4146-4147.

26. You Y, He L, Zhang M, Zhang J (2015) Comparative genomics of a Helicobacter pylori isolate from a Chinese Yunnan Naxi ethnic aborigine suggests high genetic divergence and phage insertion. PLoS One 10: e0120659.

27. Yahara K, Furuta Y, Oshima K, Yoshida M, Azuma T, et al. (2013) Chromosome painting in silico in a bacterial species reveals fine population structure. Mol Biol Evol 30: 1454-1464.

28. Wang HC, Cheng FC, Wu MS, Shu HY, Sun HS, et al. (2015) Genome Sequences of Three Helicobacter pylori Strains from Patients with Gastric Mucosa-Associated Lymphoid Tissue Lymphoma. Genome Announc 3.

29. Khosravi Y, Rehvathy V, Wee WY, Wang S, Baybayan P, et al. (2013) Comparing the genomes of Helicobacter pylori clinical strain UM032 and Mice-adapted derivatives. Gut Pathog 5: 25.

30. Monteiro MA, Zheng P, Ho B, Yokota S, Amano K, et al. (2000) Expression of histo-blood group antigens by lipopolysaccharides of Helicobacter pylori strains from Asian hosts: the propensity to express type 1 blood-group antigens. Glycobiology 10: 701-713.

31. Kersulyte D, Bertoli MT, Tamma S, Keelan M, Munday R, et al. (2015) Complete Genome Sequences of Two Helicobacter pylori Strains from a Canadian Arctic Aboriginal Community. Genome Announc 3.

32. Mane SP, Dominguez-Bello MG, Blaser MJ, Sobral BW, Hontecillas R, et al. (2010) Host-interactive genes in Amerindian Helicobacter pylori diverge from their Old World homologs and mediate inflammatory responses. J Bacteriol 192: 3078-3092.
