# Supplementary material for: East-Asian Helicobacter pylori strains synthesize heptan-deficient lipopolysaccharide
Source: PLoS Genet. 2019 Nov 20;15(11):e1008497. doi: 10.1371/journal.pgen.1008497 (PMC6892558; doi:10.1371/journal.pgen.1008497)
Supplement: S4 Table — (DOCX) [file pgen.1008497.s010.docx]

**S4 Table. List of *H. pylori* strains containing *HP1283***

| **NO.** | **Strains** | **Isolation Country** | **HP1283 homolog** | **Protein_ID** | **Assigned population** |
| --- | --- | --- | --- | --- | --- |
|  | 26695 | UK | HP_1283 | AAD08329.1 | hpEurope |
|  | G27 | Italy | HPG27_1235 | WP_000838757.1 | hpEurope |
|  | P12 | Germany | HPP12_1249 | WP_000838758.1 | hpEurope |
|  | Lithuania75 | Lithuania | HPLT_06445 | ADU83679.1 | hpEurope |
|  | Hp H-45 | USA | HPHPH45_1225 | WP_000838759.1 | hpEurope |
|  | Hp P-23 | USA | HPHPP23_1625 | EJC11105.1 | hpEurope |
|  | Hp P-74 | USA | HPHPP74_0552 | EJC17779.1 | hpEurope |
|  | Hp H-28 | USA | HPHPH28_1464 | EJB52215.1 | hpEurope |
|  | NQ4076 | Colombia | HPNQ4076_1245 | EJB33248.1 | hpEurope |
|  | NQ4099 | Colombia | HPNQ4099_1498 | EJB28274.1 | hpEurope |
|  | HP87 P7 | Germany | HP87P7_700 | N/A | hpEurope |
|  | 1846/05 | [Portugal](https://cn.bing.com/dict/search?q=Portugal&FORM=BDVSP6&mkt=zh-cn) | HPY1846_02145 | WP_042636293.1 | hpEurope |
|  | 1786/05 | [Portugal](https://cn.bing.com/dict/search?q=Portugal&FORM=BDVSP6&mkt=zh-cn) | HPY1786_06485 | KHL81314.1 | hpEurope |
|  | STT-1-1-12-Cm | Germany | BAX16_MM_358 | N/A | hpEurope |
|  | UM209 | Malaysia | ACM24_00490 | WP_050841208.1 | hpEurope |
|  | SS1 | Australia | X568_00310 | WP_000838751.1 | hpEurope |
|  | X47 | USA | N871_04215 | WP_023526569.1 | hpEurope |
|  | BM012A | Australia | U063_0323 | AHA87688.1" | hpEurope |
|  | BM012B | Australia | EG66_01715 | AHZ27908.1 | hpEurope |
|  | BM012S | Australia | U064_0324 | WP_023591695.1 | hpEurope |
|  | BM013A | Australia | EG63_06670 | WP_038418102.1 | hpEurope |
|  | BM013B | Australia | EG64_06675 | WP_038418102.1 | hpEurope |
|  | R32b | Canada | OUG_1553 | WP_000838750.1 | hpEurope |
|  | R038b | Canada | OUM_1479 | WP_000838761.1 | hpEurope |
|  | Hp P-30 | USA | HPHPP30_0744 | EJC51603.1 | hpEurope |
|  | Hp H-43 | USA | HPHPH43_1342 | EJB63282.1 | hpEurope |
|  | Hp A-27 | USA | HPHPA27_1289 | EJB75689.1 | hpEurope |
|  | NQ4053 | Colombia | HPNQ4053_1407 | EJB32769.1 | hpEurope |
|  | NQ4110 | Colombia | HPNQ4110_1453 | EJB37595.1 | hpEurope |
|  | Pecan4 | Peru | HPPC_06750 | ADO07558.1 | hpEurope |
|  | 7C | Mexico | APV63_RS06535 | WP_060473613.1 | hpEurope |
|  | 29CaP | Mexico | 29CaP_1360 | N/A | hpEurope |
|  | UMB_G1 | Canada | A607_1131 | WP_001922230.1 | hpEurope |
|  | SV376_1 | Colombia | BZK24_06370 | WP_077656915.1 | hpEurope |
|  | 228/99 | [Portugal](https://cn.bing.com/dict/search?q=Portugal&FORM=BDVSP6&mkt=zh-cn) | HPY228_06190 | WP_042633888.1 | hpEurope |
|  | 655/99 | [Portugal](https://cn.bing.com/dict/search?q=Portugal&FORM=BDVSP6&mkt=zh-cn) | HPY655_06395 | KHL85823.1 | hpEurope |
|  | 173/00 | [Portugal](https://cn.bing.com/dict/search?q=Portugal&FORM=BDVSP6&mkt=zh-cn) | HPY173_07590 | KHL81355.1 | hpEurope |
|  | 207/99 | [Portugal](https://cn.bing.com/dict/search?q=Portugal&FORM=BDVSP6&mkt=zh-cn) | HPY207_04120 | WP_039092427.1 | hpEurope |
|  | CA22337 | Colombia | B0X34_04415 | OOP85576.1 | hpEurope |
|  | CA26024 | Colombia | B0X46_07275 | OOQ02369.1 | hpEurope |
|  | CA22362 | Colombia | B0X36_00120 | WP_078283172.1 | hpEurope |
|  | A45 | Russia | C528_04995 | WP_001917199.1 | hpEurope |
|  | Hp P-15 | USA | HPHPP15_1599 | EJC06403.1 | hpEurope |
|  | Hp P-15b | USA | HPHPP15B_0591 | EJC31771.1 | hpEurope |
|  | Hp H-27 | USA | HPHPH27_0628 | EJB53998.1 | hpEurope |
|  | NCTC11637 | Australia | HP17_01458 | WP_000838760.1 | hpEurope |
|  | Aklavik117 | Canada | HPAKL117_06540 | WP_015086284.1 | hspAmerind |
|  | V225d | Venezuela | HPV225_1408 | ADI35439.1 | hspAmerind |
|  | 132 | Singapore | BHU50_04085 | WP_075651678.1 | hpAsia2 |
|  | 132A | Singapore | BIZ48_04710 | OLR46540.1 | hpAsia2 |
|  | UM087 | Malaysia | AB991_05905 | WP_048946135.1 | hpAsia2 |
|  | UM084 | Malaysia | N203_05850 | EPZ73842.1 | hpAsia2 |
|  | PNG84A | Papua New Guinea | AA974_06225 | WP_064433841.1 | hpSahul |
|  | ausabrJ05 | [Australia](https://www.ncbi.nlm.nih.gov/biosample?term=%22geo_loc_name=Jigalong,%20Australia%22%5battr%5d) | AA973_RS06170 | WP_064438028.1 | hpSahul |
|  | Sahul64 | [Australia](https://www.ncbi.nlm.nih.gov/biosample?term=%22geo_loc_name=Jigalong,%20Australia%22%5battr%5d) | HPSAHUL64_RS0108285 | WP_024751590.1 | hpSahul |
|  | GAM264Ai | Gambia | HMPREF1420_01396 | WP_025451949.1 | hpAfrica1 |
|  | GAM201Ai | Gambia | HMPREF1403_00705 | WP_025453073.1 | hpAfrica1 |

N/A, not applicable.
