# Supplementary material for: East-Asian Helicobacter pylori strains synthesize heptan-deficient lipopolysaccharide
Source: PLoS Genet. 2019 Nov 20;15(11):e1008497. doi: 10.1371/journal.pgen.1008497 (PMC6892558; doi:10.1371/journal.pgen.1008497)
Supplement: S5 Table — (DOCX) [file pgen.1008497.s011.docx]

**S5 Table**. Plasmids and bacterial strains used in this study

| **Plasmid or strain name** | **Description** | **Source / reference** |
| --- | --- | --- |
| pENT-RC | pEntranceposon, containing *rpsL-cat* flanked by MuA transposase recognition site | Ondek |
| pDifWT-RC | *rpsL-cat* cassette flanked by *H. pylori* *difH* sequence | [[1](#_ENREF_1)] |
| pHel2 | *E. coli-H. pylori* shuttle plasmid | [[2](#_ENREF_2)] |
| pHel2_uP | Derivative of pHel2 containing core urease promotor | [[3](#_ENREF_3)] |
| pCR2.1-0102 | pCR^®^2.1-TOPO^®^ vector containing sequence *HP0102* | This work |
| pCR2.1-0102-RC | pCR2.1-0102 containing the *rpsL-cat* insertion within the sequence *HP0102* | This work |
| pHel2_uP_HP0102 | pHel2_uP expressing *HP0102* | This work |
| p0159-AB | pGEM^®^-T Easy vector containing sequences flanking *HPG27_146*, separated by a *Bam*HI restriction site | This work |
| p0159-AB-difH-RC | Derivative of p0159-AB, with *difH-rpsL-cat-difH* inserted between *HPG27_146* flanking sequences at the *Bam*HI site | This work |
| p1105-AB | pGEM^®^-T Easy vector containing sequences flanking *HPG27_1046*, separated by a *Bam*HI restriction site | This work |
| p1105-AB-difH-RC | Derivative of p1105-AB, with *difH-rpsL-cat-difH* inserted between *HPG27_1046* flanking sequences at the *Bam*HI site | This work |
| p1416-AB | pGEM^®^-T Easy vector containing sequences flanking *HPG27_1339*, separated by a *Bam*HI restriction site | This work |
| p1416-AB-difH-RC | Derivative of p1416-AB, with *difH-rpsL-cat-difH* inserted between *HPG27_1339* flanking sequences at the *Bam*HI site | This work |
| p0208-AB | pGEM^®^-T Easy vector containing sequences flanking *HPG27_190*, separated by a *Bam*HI restriction site | This work |
| p0208-AB-difH-RC | Derivative of p0579-AB, with *difH-rpsL-cat-difH* inserted between *HPG27_190* flanking sequences at the *Bam*HI site | This work |
| p1578-AB | pGEM^®^-T Easy vector containing sequences flanking *HPG27_1515*, separated by a *Bam*HI restriction site | This work |
| p1578-AB-difH-RC | Derivative of p1578-AB, with *difH-rpsL-cat-difH* inserted between *HPG27_1515* flanking sequences at the *Bam*HI site | This work |
| pHel2_uP_HP1578 | pHel2_uP expressing *HP1578* | This work |
| p0479-AB | pGEM^®^-T Easy vector containing sequences flanking *HPG27_437*, separated by a *Bam*HI restriction site | This work |
| p0479-AB-difH-RC | Derivative of p0479-AB, with *difH-rpsL-cat-difH* inserted between *HPG27_437* flanking sequences at the *Bam*HI site | This work |
| p0279-AB | pGEM^®^-T Easy vector containing sequences flanking *HPG27_258*, separated by a *Bam*HI restriction site | This work |
| p0279-AB-difH-RC | Derivative of p0279-AB, with *difH-rpsL-cat-difH* inserted between *HPG27_258* flanking sequences at the *Bam*HI site | This work |
| p1191-AB | pGEM^®^-T Easy vector containing sequences flanking *HPG27_1136*, separated by a *Bam*HI restriction site | This work |
| p1191-AB-difH-RC | Derivative of p0479-AB, with *difH-rpsL-cat-difH* inserted between *HPG27_1136* flanking sequences at the *Bam*HI site | This work |
| pCR2.1-1283 | pCR^®^2.1-TOPO^®^ vector containing sequence *HP1283* | This work |
| pCR2.1-1283-RC | pCR2.1-1283 containing the *rpsL-cat* insertion within the sequence *HP1283* | This work |
| pHel2_uP_HP1283 | pHel2_uP expressing *HP1283* | This work |
| pG27_1230-AB | pGEM^®^-T Easy vector containing sequences flanking *HPG27_1230*, separated by a *Bam*HI restriction site | This work |
| pG27_1230-AB-difH-RC | Derivative of HPG27_1230-AB, with *difH-rpsL-cat-difH* inserted between *HPG27_1230* flanking sequences at the *Bam*HI site | This work |
| pFutA-AB | pGEM^®^-T Easy vector containing sequences flanking *HPG27_613*, separated by a *Bam*HI restriction site | This work |
| pFutA-AB-difH-RC | Derivative of pFutA-AB, with *difH-rpsL-cat-difH* inserted between *HPG27_613* flanking sequences at the *Bam*HI site | This work |
| pFutB-AB | pGEM^®^-T Easy vector containing sequences flanking *HPG27_1018*, separated by a *Bam*HI restriction site | This work |
| pFutB-AB-difH-RC | Derivative of pFutB-AB, with *difH-rpsL-cat-difH* inserted between *HPG27_1018* flanking sequences at the *Bam*HI site | This work |
| pFutC-AB | pGEM^®^-T Easy vector containing sequences flanking *HPG27_86*, separated by a *Bam*HI restriction site | This work |
| pFutC-AB-difH-RC | Derivative of pFutC-AB, with *difH-rpsL-cat-difH* inserted between *HPG27_86* flanking sequences at the *Bam*HI site | This work |
| p0826-AB | pGEM^®^-T Easy vector containing sequences flanking *HPG27_785*, separated by a *Bam*HI restriction site | This work |
| p0826-AB-difH-RC | Derivative of p0826-AB, with *difH-rpsL-cat-difH* inserted between *HPG27_785* flanking sequences at the *Bam*HI site | This work |
| p0619-AB | pGEM^®^-T Easy vector containing sequences flanking *HPG27_579/580*, separated by a *Bam*HI restriction site | This work |
| p0619-AB-difH-RC | Derivative of p0619-AB, with *difH-rpsL-cat-difH* inserted between *HPG27_579/580* flanking sequences at the *Bam*HI site | This work |
| pCR2.1-0805 | pCR^®^2.1-TOPO^®^ vector containing sequence *HP0805* | This work |
| pCR2.1-0805-RC | pCR2.1-0805 containing the *rpsL-cat* insertion within the sequence *HP0805* | This work |
| pWecA-AB | pGEM^®^-T Easy vector containing sequences flanking *HPG27_1518*, separated by a *Bam*HI restriction site | This work |
| pWecA-AB-difH-RC | Derivative of pWecA-AB, with *difH-rpsL-cat-difH* inserted between *HPG27_1518* flanking sequences at the *Bam*HI site | This work |
| pWzk-AB | pGEM^®^-T Easy vector containing sequences flanking *HPG27_1153*, separated by a *Bam*HI restriction site | This work |
| pWzk-AB-difH-RC | Derivative of pWzk-AB, with *difH-rpsL-cat-difH* inserted between *HPG27_1153* flanking sequences at the *Bam*HI site | This work |
| ***H. pylori* strains** |  |  |
| G27^Str^ | Streptomycin resistant | [[4](#_ENREF_4)] |
| G27Δ*HP0102* | Clean deletion of *HPG27_94* in G27 | This work |
| G27Δ*HP0102* *comp* | The conjugation of pHel2_uP_HP0102 into G27Δ*HP0102* | This work |
| G27Δ*HP0159* | Clean deletion of *HPG27_146* in G27 | This work |
| G27Δ*HP1105* | Clean deletion of *HPG27_1046* in G27 | This work |
| G27Δ*HP1416* | Clean deletion of *HPG27_ 1339* in G27 | This work |
| G27Δ*HP0208* | Clean deletion of *HPG27_190* in G27 | This work |
| G27Δ*HP1578* | Clean deletion of *HPG27_1515* in G27 | This work |
| G27Δ*HP0479* | Clean deletion of *HPG27_437* in G27 | This work |
| G27Δ*HP1191* | Clean deletion of *HPG27_1136* in G27 | This work |
| G27Δ*HP1283* | Clean deletion of *HPG27_1235* in G27 | This work |
| G27Δ*HP1283* *comp* | The conjugation of pHel2_uP_HP1283 into G27Δ*HP1283* | This work |
| G27Δ*HP1284* | Clean deletion of *HPG27_1236* in G27 | [[3](#_ENREF_3)] |
| G27Δ*HPG27_1230* | Clean deletion of *HPG27_1230* in G27 | This work |
| G27Δ*futA* | Clean deletion of *HPG27_613* in G27 | This work |
| G27Δ*futB* | Clean deletion of *HPG27_1018* in G27 | This work |
| G27Δ*futC* | Clean deletion of *HPG27_86* in G27 | This work |
| G27Δ*HP0826* | Clean deletion of *HPG27_785* in G27 | This work |
| G27Δ*HP0619* | Clean deletion of *HPG27_579/580* in G27 | This work |
| G27Δ*HP0805* | Clean deletion of *HPG27_761* in G27 | This work |
| G27Δ*wecA* | Clean deletion of *HPG27_1518* in G27 | This work |
| G27Δ*wzk* | Clean deletion of *HPG27_1153* in G27 | This work |
| G27Δ*waaL* | Clean deletion of *HPG27_389* in G27 | [[3](#_ENREF_3)] |
| ***E. coli* strains** |  |  |
| β2150 donor strain | Δ*dapA*, erm^r^ | [[2](#_ENREF_2)] |
| β2150 helper strain | Δ*dapA*, kan^r^, erm^r^ | [[2](#_ENREF_2)] |

**References**

1. Debowski AW, Carnoy C, Verbrugghe P, Nilsson HO, Gauntlett JC, et al. (2012) Xer recombinase and genome integrity in *Helicobacter pylori*, a pathogen without topoisomerase IV. PLoS One 7: e33310.
2. Heuermann D, Haas R (1998) A stable shuttle vector system for efficient genetic complementation of *Helicobacter pylori* strains by transformation and conjugation. Mol Gen Genet 257: 519-528.
3. Li H, Yang T, Liao T, Debowski AW, Nilsson HO, et al. (2017) The redefinition of *Helicobacter pylori* lipopolysaccharide O-antigen and core-oligosaccharide domains. PLoS Pathog 13: e1006280.
4. Baltrus DA, Amieva MR, Covacci A, Lowe TM, Merrell DS, et al. (2009) The complete genome sequence of *Helicobacter pylori* strain G27. J Bacteriol 191: 447-448.
