# Supplementary material for: East-Asian Helicobacter pylori strains synthesize heptan-deficient lipopolysaccharide
Source: PLoS Genet. 2019 Nov 20;15(11):e1008497. doi: 10.1371/journal.pgen.1008497 (PMC6892558; doi:10.1371/journal.pgen.1008497)
Supplement: S6 Table — (DOCX) [file pgen.1008497.s012.docx]

**S6 Table.** Oligonucleotides used in this study

| **Primer set** | **Sequence (5'--> 3')** | **Function of PCR product** |
| --- | --- | --- |
| **HP0102** |  |  |
| HP 0102-F | GGTAAGCGATTTTGCAGAGGTG | Construction of pCR2.1-0102 |
| HP 0102-R | CACCTACAACAACCATCAAGAAAG |  |
| **HP0102 SOE** |  |  |
| HP0102 clean KO-F1 | GTGGGTGATCGCAACGATGAAATC | Clean deletion of *HP0102* |
| HP0102 clean KO-F2 | GAGCATGAAAGATTCCTCAACC |  |
| HP0102 clean KO-F3 | GTTTATTAAAAAAGGGTTGGACTAAAACGAAACGATTAAAGAAAG |  |
| HP0102 clean KO-R1 | GCTTGATGGGAACAAAACCTC |  |
| HP0102 clean KO-R2 | CACTTGGTCTAAATTCTTGG |  |
| HP0102 clean KO-R3 | CGTTTCGTTTTAGTCCAACCCTTTTTTAATAAACTCCCCCTGATTAAG |  |
| **HP0102 Comp** |  | Construction of pHel2_HP0102 |
| HPG27_94 XhoI-F | GACGACCTCGAGTTGTTAAAAGTTTCTGTGATCACG |  |
| HPG27_94 XhoI-R | GTCGTCCTCGAGTTAATCGTTTCGTTTTAATCC |  |
| **HP0159** |  | Construction of p0159-AB-difH-RC |
| HP0159-F | CACCTTACAGCTACCACTAAAGC |  |
| HP0159-BamHI-R | GAACGCTAAGAGGATCCTTTAAGTAGTTTTCCTAAAATAGG |  |
| HP0159-BamHI-F | CTACTTAAAGGATCCTCTTAGCGTTCTCGTTTGGGCAAC |  |
| HP0159-R | GCACCGAGTTATACGCTTATC |  |
|  | GATGGCTGGCGTTATGAAAAACG |  |
| **HP1105** |  | Construction of p1105-AB-difH-RC |
| HP1105-F | GATGGCTGGCGTTATGAAAAACG |  |
| HP1105-BamHI-R | GCTTTTTACTGGATCCAAAAAATTAAACACCCTTTTTTAATGG |  |
| HP1105-BamHI-F | GTTTAATTTTTTGGATCCAGTAAAAAGCCTTTAATCAAACG |  |
| HP1105-R | CCTAAAGATGTGCTGATTGAC |  |
| **HP1416** |  | Construction of p1416-AB-difH-RC |
| HP1416-F | GAAACTCATCGCGCTTTTAGG |  |
| HP1416-BamHI-R | GTTTTTTTTGTCCTGGATCCTAGATTACCCTTTAATACCGCTTTTTGAG |  |
| HP1416-BamHI-F | GGTAATCTAGGATCCAGGACAAAAAAAACGATGAAATTTTTAAAATTC |  |
| HP1416-R | CCAAGGCTTAGTCGTTTCAGC |  |
| **HP0208** |  | Construction of p0208-AB-difH-RC |
| HP0208-F | CCAAAGGTGCTAAACCACAC |  |
| HP0208-BamHI-R | GAAAGGGGGTGGATCCACAATGGATTTGATAAAAGAGTTTTACTC |  |
| HP0208-BamHI-F | CAAATCCATTGTGGATCCACCCCCTTTCAAAACTAATGCGAGCAAGC |  |
| HP0208-R | GCTTTTGGCGTTTCTGTGATG |  |
| **HP1578** |  | Construction of p1578-AB-difH-RC |
| HP1578-F | GAGATTGGCGATGTGCTTAGG |  |
| HP1578-BamHI-R | GATTCCAACAAAGGATCCCAATTTTCCTTGGAGATTTTGTG |  |
| HP1578-BamHI-F | GAAAATTGGGATCCTTTGTTGGAATCACAAGGCGAAGC |  |
| HP1578-R | GCAAGACGTTTATCGCATCGTG |  |
| **HP1578 Comp** |  | Construction of pHel2_HP1578 |
| HPG27_1515 XhoI-F | GACGACCTCGAGATGCAACATGAAATCCCTATTGC |  |
| HPG27_1515 XhoI-R | GTCGTCCTCGAGTTAGAACTTTTTTTGAAAAAATTTTTTTTG |  |
| **HP0479** |  | Construction of p0479-AB-difH-RC |
| HP0479-F | GCTCATTTGGGGCTATTATTACC |  |
| HP0479-BamHI-R | GTATAACACAATAGGATCCATGAGTGCCTAGGATTTTAAGGGGTTGTTG |  |
| HP0479-BamHI-F | CTAGGCACTCATGGATCCTATTGTGTTATACTTCTAATTTCAATTTTGC |  |
| HP0479-R | CACGCTCATCGTAGGCTCTTC |  |
| **HP1191** |  | Construction of p1191-AB-difH-RC |
| HP1191-F | CACACGCTCCCTAAAGACTTC |  |
| HP1191-BamHI-R | CCATAAAAGGCGGGATCCCAATAGCCCTTTTTTAAAGATTTTAAGC |  |
| HP1191-BamHI-F | CTTTAAAAAAGGGCTATTGGGATCCCGCCTTTTATGGGCTTTTTTAAAAACAC |  |
| HP1191-R | CCATGCACCCTAGAGCTAATGC |  |
| **HP0279** |  | Construction of p0279-AB-difH-RC |
| HP0279-F | GGACTTAATCTCGCTTGATGTTGG |  |
| HP0279-BamHI-R | CTTTGTAAGGGATCCTTTCAAGCAAACTCTATCGTCAAAGG |  |
| HP0279-BamHI-F | GAGTTTGCTTGAAAGGATCCCTTACAAAGAACGACTCATACACG |  |
| HP0279-R | GTGATTTGAGAGCGGATACTTTC |  |
| **HP1283** |  | Construction of pCR2.1-1283 |
| HP1283-F | GCGTGGATTTTACATTGTTGAACAC |  |
| HP1283-R | GGTTGTTTCGTTGGTATTAGATG |  |
| **HP1283 SOE** |  | Clean deletion of *HP1283* |
| HP1283 clean KO-F1 | GACAACCATTTGAGCGCCACTCTC |  |
| HP1283 clean KO-F2 | CCAGACTCGCTTTTAGTTTGAGG |  |
| HP1283 clean KO-F3 | GGAGCTGTTTTTGCGTTATAAGTTTTTAGAGGATAACTTCAGC |  |
| HP1283 clean KO-R1 | CTTCTTGTAAGGATAGCACTC |  |
| HP1283 clean KO-R2 | GTCATGCACTGTGTTGTCTTTCG |  |
| HP1283 clean KO-R3 | CTTATAACGCAAAAACAGCTCCTATTTTTTAGTGTTCAAC |  |
| **HP1283 Comp** |  | Construction of pHel2_HP1283 |
| HP G27_1235 XhoI-F | GACGACCTCGAGTTGAAGTCTTTACTCTCTTTG |  |
| HP G27_1235 XhoI-R | GTCGTCCTCGAGTCAAACTCGTTCAAAATAGGTTG |  |
| **HPG27_1230** |  | Construction of pG27_1230-AB-difH-RC |
| HPG27_1230-F | CGTCAAAAGGGCTAGGTTCAAAG |  |
| HPG27_1230-BamHI-R | GTTTGGACTGCTGGATCCTTCAAAGAGCCTTAAAGACCAATCAG |  |
| HPG27_1230-BamHI-F | CTTTGAAGGATCCAGCAGTCCAAACAACGCCATC |  |
| HPG27_1230-R | CCAGCCTAAAAGAGCGTTATTATGC |  |
| **FutA** |  | Construction of pFutA-AB-difH-RC |
| FutA-F | GTGCCTGTGCAATTGACTAG |  |
| FutA-BamHI-R | CTTAAAATACGCCGGATCCGGCTATCCTTTAAAGGAGTATTTTAC |  |
| FutA-BamHI-F | GGATAGCCGGATCCGGCGTATTTTAAGACTGATTAAG |  |
| FutA-R | GGTTATCGCTTACAAGCTTTTCC |  |
| **FutB** |  |  |
| FutB-F | GCAACGCTTATGAGTCCATGC | Construction of pFutB-AB-difH-RC |
| FutB-BamHI-R | GTTTTACAAACGGATCCCTTTATCTTTTAAACTAATTTAGGCAAAC |  |
| FutB-BamHI-F | GTTTAAAAGATAAAGGGATCCGTTTGTAAAACAAACCCATCAAACC |  |
| FutB-R | CCAGCTGGGGATATTGGAGTG |  |
| **FutC** |  | Construction of pFutC-AB-difH-RC |
| FutC-F | CCTGTAGCGGTGATGCAAATGAG |  |
| FutC-BamHI-R | CCTTTTTTAAGCCGCTGGATCCTCAATCCCTTTATATTGTCAAATAAAACC |  |
| FutC-BamHI-F | CAATATAAAGGGATTGAGGATCCAGCGGCTTAAAAAAGGGCTTATTGG |  |
| FutC-R | CACAGGTTGGTTGGACGCTAAAAAC |  |
| **HP0826** |  | Construction of p0826-AB-difH-RC |
| HP0826-F | GCAAGAGCAATGCTTTCGCTTTG |  |
| HP0826-BamHI-R | GTATTTTTATTATTGAGGATCCAGCTTAGACTCCCATAATTTTTAATAG |  |
| HP0826-BamHI-F | GTCTAAGCTGGATCCTCAATAATAAAAATACTAAAGAGC |  |
| HP0826-R | GGATCGTGTCTTTATCGTTTCG |  |
| **HP0619** |  | Construction of p0619-AB-difH-RC |
| HPG27_579/580-F | GCGTATGATGTGGTGCTTAATGG |  |
| HPG27_579/580-BamHI-R | CGCTCTTTGGATCCAGCTCATTCTCTTTGGAACG |  |
| HPG27_579/580-BamHI-F | GAATGAGCTGGATCCAAAGAGCGGGCTTTATGTTAGAATAAGC |  |
| HPG27_579/580-R | CCATCCCCAAACCTAAAGAAGC |  |
| **HP0805** |  |  |
| HP0805-F | CTCGCTCTAGTTTGAGAGCAAG | Construction of pCR2.1-0805 |
| HP 0805-R | GAAACTCCCCCTATCATGCAAG |  |
| **HP0805 SOE** |  | SOE PCR for clean deletion of *HP0805* |
| HP0805 clean KO-F1 | GGTGCTATCACTACCAAAACAGC |  |
| HP0805 clean KO-F2 | CTAGGAGCATCACAGCGTATGTG |  |
| HP0805 clean KO-F3 | GAATTAAGGCTAACAATAAGTCTTTGAGCATTTCTTGCATG |  |
| HP0805 clean KO-R1 | CACTTCAAACGCATCAGTCG |  |
| HP0805 clean KO-R2 | GTGTAGCATTGCGTTTGGTG |  |
| HP0805 clean KO-R3 | CTCAAAGACTTATTGTTAGCCTTAATTCTTAATGCAAATAAAATC |  |
| **WecA** |  | Construction of pWecA-AB-difH-RC |
| WecA-F | CACGCTATGACCGATATTAAGC |  |
| WecA-BamHI-R | GCTCGCTTTTCTAAGGATCCCCACAACACTTTTTATTGGATTTAATTGG |  |
| WecA-BamHI-F | GTGGGGATCCTTAGAAAAGCGAGCGTTTCAATG |  |
| WecA-R | GCTGTTCTGTTTGAGACAAG |  |
| **Wzk** |  | Construction of pWzk-AB-difH-RC |
| Wzk-F | GTGAGAAGTAGCGATAGTGATC |  |
| Wzk-BamHI-R | GAGTAAAGCCAACTGGATCCAATTTAAGTGTCCATAAATTC |  |
| Wzk-BamHI-F | CACTTAAATTGGATCCAGTTGGCTTTACTCTTTTATAAAG |  |
| Wzk-R | GGTGCTTACTCCTTAATGACC |  |
