# Supplementary material for: East-Asian Helicobacter pylori strains synthesize heptan-deficient lipopolysaccharide
Source: PLoS Genet. 2019 Nov 20;15(11):e1008497. doi: 10.1371/journal.pgen.1008497 (PMC6892558; doi:10.1371/journal.pgen.1008497)
Supplement: S1 Text — (DOCX) [file pgen.1008497.s013.docx]

**S1 Text**

**Systematic construction of *H. pylori* LPS mutants and complementation**

At the time of constructing *HP0805*, *HP0102* and *HP1283* mutants, the whole genome sequence of G27 strain was not available, and therefore ORFs *HP1283, HP0102* and *HP0805* were amplified from 26695 genomic DNA using primers HP1283F and HP1283R, HP0102F and HP0102R, HP0805F and HP0805R. These PCR products were then individually TA-cloned into the pCR^®^2.1-TOPO^®^ vector to give pCR2.1-1283, pCR2.1-0102 and pCR2.1-0805. The *rpsL-cat* cassette was liberated from pENT-RC by *Bgl*II digest and inserted into above-constructed pCR2.1 derivatives using MuA transposase *in vitro* transposition to give pCR2.1-1283-RC, pCR2.1-0102-RC and pCR2.1-0805-RC, which were then used to transform strain G27 to generate G27*HP1283::RC*, G27*HP0102::RC*, G27*HP0805::RC.* Using primer set HP1283, HP0102 and HP0805 (**S2 Table**), clean deletions of the *HP1283, HP0102* and *HP0805* in G27 was achieved as previously described [[1](#_ENREF_1)], generating the corresponding mutants G27Δ*HP1283*, G27Δ*HP0102* and G27Δ*HP0805* (**S1 Table**)*.*

**The systematic deletion of other LPS genes in G27**. G27 mutants other than *HP0805*, *HP0102* and *HP1283* (**S1 Table**) in this study were constructed using a highly efficient Xer-cise gene deletion method [[1](#_ENREF_1)]. For the construction of Δ*HP0159* mutant, primer set HP0159 was used (**S2 Table**). Two DNA fragments were amplified from G27 genomic DNA using primers HP0159-F and HP0159-BamHI-R, and HP0159-BamHI-F and HP0159-R. The two DNA fragments were joined by SOE PCR to give a 1.9-kb fragment containing the DNA regions flanking *HPG27_146* separated by a unique *Bam*HI site. This PCR product was treated with *MyTaq* DNA polymerase and ligated into pGEM^®^-T Easy Vector to give p0159-AB. The *difH* flanked *rpsL-cat* cassette was liberated from pDifWT-RC by *Bam*HI digest and cloned into the unique *Bam*HI site of p0159-AB to give p0159-AB-difH-RC, which was used to transform strain G27 to generate G27Δ*HP0159*.

Similar to the construction of Δ*HP0159* mutant, primer sets HP1105, HP1416, HP0208, HP1578, HP0479, FutA, FutB, FutC, WecA, Wzk (**S2 Table**) were used to successfully construct corresponding mutants except Δ*HP0279*. Of note, plasmid p0279-AB-difH-RC was successfully constructed, whereas numerous attempts using this plasmid to transform strain G27 to generate G27Δ*HP0279* were failed.

**The complementation of G27Δ*HP1283*, G27Δ*HP0102* and G27Δ*HP1578***. *HPG27_1235* (corresponds to *HP1283*), *HPG27_94* (corresponds to *HP0102*) and *HPG27_1515* (corresponds to *HP1578*) were amplified from G27 genomic DNA using primer sets HP1283 Comp, HP0102 Comp and HP1578 Comp, respectively. The PCR products were digested with *Xho*I and cloned into the unique *Xho*I site of pHel2_uP to give pHel2_uP_HP1283, pHel2_uP_HP0102 and pHel2_uP_HP1578, respectively. The correct orientation of the genes was confirmed by successful amplification of the PCR products - HP1283 - 1.5 kb PCR product using primers HP G27_1235 XhoI-F and pHel2F, HP0102 - 1.0 kb PCR product using primers HPG27_94 XhoI-F and pHel2F and HP1578 - 1.2 kb PCR product using primers HPG27_1515 XhoI-F and pHel2F. The plasmids pHel2_uP_HP1283 and pHel2_uP_HP0102 were successfully introduced into G27Δ*HP1283* and G27Δ*HP0102* respectively by conjugation in a tri-parental mating format as previously described [[2](#_ENREF_2)]. However, several attempts to introduce the complementation plasmid pHel2_uP_HP1578 into G27Δ*HP1578* were unsuccessful.

**LPS Structural Analysis**

**Methanolysis and mild HF hydrolysis.** LPS samples were dissolved in 0.5 M methanolic-HCl solution and incubated at 50°C for 30-60 min before dried under a stream of nitrogen. The products were permethylated and analysed by MALDI-TOF and TOF/TOF. For HF hydrolysis, LPS samples were hydrolysed as previously described [[3](#_ENREF_3)] with minor modifications. Briefly, the samples were dissolved in 50 μL of 48% HF and incubated at 4°C for 24 h. The reagents were removed under a stream of nitrogen.

**Mild periodate oxidation.** The LPS sample was dissolved in 100 μL sodium periodate solution (20 mM, in 100 mM ammonium acetate buffer, pH = 6.5) and incubated at 4°C for 20 h in the dark. A few drops of ethylene glycol were added, and the mixture was kept at room temperature for 1 h to terminate the reaction. Sodium borohydride (400 μL, 10 mg/mL in 2 M ammonium hydroxide) was then added to the solution and incubated at room temperature for 2 h. The reaction was quenched by adding 3-5 drops of acetic acid, and the products were purified by Dowex resin.

**Permethylation.** Pellets of sodium hydroxide (3-5 per sample) were crushed in dimethyl sulfoxide (3 mL). The resulting slurry (0.75 mL) and iodomethane (0.85 mL) were added to each sample. The mixture was agitating at room temperature (60 min) before the reaction was quenched by adding ultrapure water (2 mL). Permethylated glycans and lipo-glycans were extracted by chloroform (2 mL) and washed with ultrapure water for two times. The chloroform was then removed under a stream of nitrogen.

**Mass spectrometry.** MS spectra were recorded by either a Voyager DE-STRTM MALDI–TOF or a 4800 MALDI-TOF/TOF mass spectrometer (Applied Biosystems, Darmstadt, Germany). All MALDI-TOF/TOF spectra were recorded by the latter instrument. MS mode was calibrated with the 4700 Calibration standard kit (Applied Biosystems) and MS/MS mode was calibrated with fibrinopeptide B (Sigma). The collision energy for TOF/TOF was set to 1 kV, and the collision gas was argon. Permethylated samples were dissolved in 10 μL methanol. A 1 μL aliquot of the solution was premixed with 1 μL of either 2, 5-Dihydroxybenzoic acid or 3,4-diaminobenzophenone matrix (20 mg/mL) before spotted an MALDI plate.

**Micelle NMR**

LPS sample (6.5 mg) was mixed with D_38_-DPC (Cambridge Isotope Laboratories. Inc.) at an estimated molar ratio of 1:40 dissolved in deuterated potassium phosphate (50 mM, pD = 6), and transferred to a 5 mm NMR tube. 1- and 2D TOCSY and NOESY NMR spectra were recorded at 30°C using a Bruker Avance III 600MHz NMR spectrometer equipped with a TXI/TCI cryoprobe.

**Accession numbers**

The whole genomes of the 45 newly sequenced strains in this study have been submitted to NCBI GenBank with the following accession numbers: CHL1(QBAB00000000), CHL2(QBAC00000000), CHL3(QBAD00000000), CHL4(QBAE00000000), CHL5(QBAF00000000), CHL6(QBAG00000000), CHL7(QBAH00000000), CHL8(QBAI00000000), CHL9(QBAJ00000000), CHL10(QBAK00000000), CHL11(QBAL00000000), CHL12(QBAM00000000), CHL14(QBAN00000000), CHL16(QBAO00000000), CHL17(QBAP00000000), CHL19(QBAQ00000000), CHL20(QBAR00000000), CHL21(QBAS00000000), CHL22(QBAT00000000), CHL23(QBAU00000000), CHL24(QBAV00000000), CHL25(QBAW00000000), CHL26(QBAX00000000), CHL27(QBAY00000000), CHL29(QBAZ00000000), CHL31(QBBA00000000), CHL32(QBBB00000000), CHL33(QBBC00000000), CHL35(QBBD00000000), CHL36(QBBE00000000), CHL37(QBBF00000000), CHL38(QBBG00000000), CHL39(QBBH00000000), CHL41(QBBI00000000), CHL42(QBBJ00000000), CHL44(QBBK00000000), CHL46(QBBL00000000), CHL47(QBBM00000000), CHL48(QBBN00000000), CHL49(QBBO00000000), CHL50(QBBP00000000), CHL51(QBBQ00000000), CHL52(QBBR00000000), CHL54(QBBS00000000), CA2(VTVC00000000).

**Reference**

1. Debowski AW, Gauntlett JC, Li H, Liao T, Sehnal M, Nilsson HO*, et al.* Xer-cise in *Helicobacter pylori*: one-step transformation for the construction of markerless gene deletions. Helicobacter 2012;**17**:435-43.
2. Heuermann D, Haas R. A stable shuttle vector system for efficient genetic complementation of *Helicobacter pylori* strains by transformation and conjugation. Mol Gen Genet 1998;**257**:519-28.
3. Haslam SM, Khoo KH, Houston KM, Harnett W, Morris HR, Dell A. Characterisation of the phosphorylcholine-containing N-linked oligosaccharides in the excretory-secretory 62 kDa glycoprotein of *Acanthocheilonema viteae*. Mol Biochem Parasitol 1997;**85**:53-66.
